# Catalytic pyrolysis mechanism of lignin moieties driven by aldehyde, hydroxyl, methoxy, and allyl functionalization: the role of reactive quinone methide and ketene intermediates†

**DOI:** 10.1039/d4gc03143a

**Published:** 2024-08-30

**Authors:** Zeyou Pan, Xiangkun Wu, Andras Bodi, Jeroen A. van Bokhoven, Patrick Hemberger

**Affiliations:** a Paul Scherrer Institute 5232 Villigen Switzerland patrick.hemberger@psi.ch; b Institute for Chemical and Bioengineering, Department of Chemistry and Applied Biosciences, ETH Zurich 8093 Zurich Switzerland

## Abstract

The catalytic pyrolysis of guaiacol-based lignin monomers, vanillin, syringol, and eugenol over commercial HZSM-5 has been investigated using *operando* Photoelectron Photoion Coincidence (PEPICO) spectroscopy to unveil the reaction mechanism by detecting reactive intermediates, such as quinone methides and ketenes, and products. *Vanillin* shares the decomposition mechanism with guaiacol due to prompt and efficient decarbonylation, which allows us to control this reaction leading to a phenol selectivity increase by switching to a faujasite catalyst and decreasing the Si/Al ratio. *Syringol* first demethylates to 3-methoxycatechol, which mainly dehydroxylates to *o*- and *m*-guaiacol. Ketene formation channels over HZSM-5 are less important here than for guaiacol or vanillin, but product distribution remains similar. C_3_ addition to guaiacol yields *eugenol*, which shows a more complex product distribution upon catalytic pyrolysis. By analogies to monomers with simplified functionalization, namely allylbenzene, 4-allylcatechol, and 4-methylcatechol, the eugenol chemistry could be fully resolved. Previously postulated reactive semi-quinone intermediates are detected spectroscopically, and their involvement opens alternative pathways to condensation and phenol formation. Allyl groups, produced by dehydroxylation of the β-O-4 bond, may not only decompose *via* C1/C2/C3 loss, but also cyclize to indene and its derivatives over HZSM-5. This comparably high reactivity leads to an unselective branching of the chemistry and to a complex product distribution, which is difficult to control. Indenes and naphthalenes are also prototypical coke precursors efficiently deactivating the catalyst. We rely on these mechanistic insights to discuss strategies to fine-tune process conditions to increase the selectivities of desired products by enhancing either vanillin and guaiacol or supressing eugenol yields from native lignin.

## Abstract 2

The complex catalytic pyrolysis mechanism of key lignin monomers demonstrates how functional groups dictate product pathways, revealing insights to enhance desired chemical yields and minimize catalyst deactivation.

## Graphical abstract

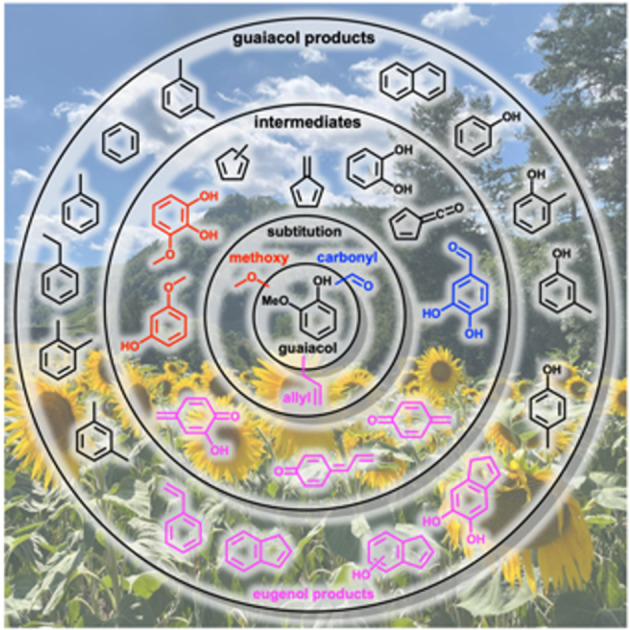

## 1. Introduction

**Scheme 1:**
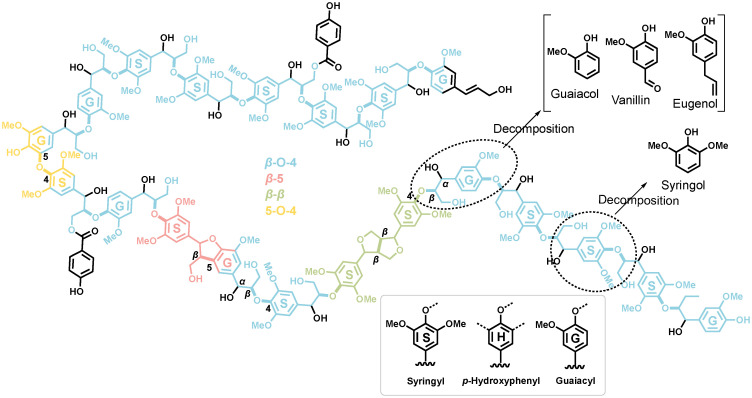
Model structure of hardwood lignin. Syringyl, *p*-hydroxyphenyl and guaiacyl units are labelled as S, H, and G in the benzene ring, respectively.^7^

Biomass, as low CO_2_-emission energy carrier, should be promoted in energy-intensive industries to slow down the global warming, according to The Intergovernmental Panel on Climate Change (IPCC).^1^ Lignin, one of main components of biomass, consists of aromatic units and is an abundant raw material for fuels and fine chemicals production *via* pyrolysis or other technologies. Various companies operating worldwide, such as Anellotech, BioBTX, Twence and Purcell, have demonstrated the technical feasibility of lignin valorization.^2–4^ However, due to the amorphous and polymeric structure of lignin, the selectivity control towards targeted products in catalytic fast pyrolysis (CFP) poses significant challenges. As a result, product selectivities are low.^5–7^ According to NMR studies, the lignin structure consists of aromatic units (guaiacyl, syringyl, and *p*-hydroxyphenyl) connected *via* different linkages (β-O-4, β-5, β–β, *etc.*), as shown in Scheme 1.^7^ Thus, a natural strategy is to investigate representative smaller model compounds, such as phenol, guaiacol, vanillin, which mimic the main lignin functionalities, to understand the reaction mechanism of specific lignin features in a bottom-up approach. This approach simplifies the number of reactions and helps unequivocally trace product formation routes.

**Scheme 2:**
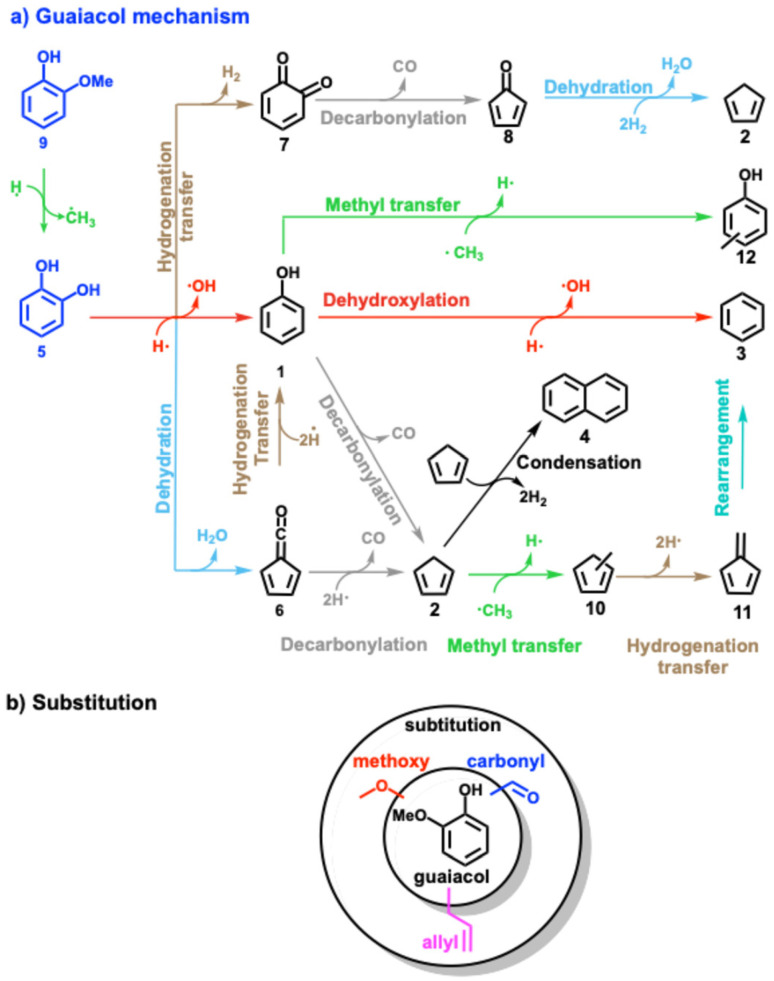
(a) Overview of the reaction mechanism of the catalytic fast pyrolysis of guaiacol, catechol, phenol over H-ZSM-5 catalyst. The colour code for each reaction (dehydroxylation, methyl transfer, *etc.*) is consistent throughout the manuscript. (b) Overview of three substituted guaiacols investigated in this study.

Lignin model compounds, such as phenol,^8–11^ anisole,^12–14^ catechol,^9–11,15,16^ guaiacol,^17–22^ and vanillin,^16,23^ were extensively studied and numerous (catalytic) pyrolysis reaction routes could be unveiled using synchrotron as well as lab-based tools.^24^ With the advent of *operando* photoelectron photoion coincidence (PEPICO) spectroscopy and other mass spectrometric methods,^25,26^ reactive intermediates could be detected, thereby probing elementary reaction steps directly. Phenol 1 (Scheme 2) represents the central aromatic structure in lignin and is a major decomposition product during pyrolysis. Experiments and calculations indicate that the unimolecular decomposition of phenol is initiated by tautomerization to cyclohexadienone, which subsequently decarbonylates to cyclopentadiene 2.^8,9^

Upon addition of a zeolite catalyst, such as HZSM-5, the newly introduced Brønsted acid sites promote phenol 1 dehydroxylation to benzene 3. As cyclopentadiene 2 undergoes rapid Diels–Alder dimerization and subsequent hydrogen transfer yielding naphthalene 4 (Scheme 2), it becomes a minor product itself.^10,11^ In addition, phenol may form diphenyl ether and naphthalene in rich gas-phase mixtures.^11^ Compared to phenol, catechol 5 has an additional hydroxyl group in the *ortho* position. Besides dehydroxylation, the two vicinal hydroxyl groups allow for a favourable transition state for intramolecular dehydration to fulvenone ketene 6.^9,15,16^ This channel is also the most relevant one over HZSM-5, albeit at a much lower light-off temperature. Intramolecular dehydration is not observed in the other two benzenediol isomers, resorcinol and hydroquinone.^10^ Over Brønsted acid sites, fulvenone 6 (Scheme 2) can decarbonylate to form cyclopentadiene 2 or can be hydrogenated to phenol 1.^10^ In a third catechol conversion pathway, dehydrogenation to *ortho*-quinone 7 is responsible for the formation of cyclopentadienone 8, which may also yield cyclopentadiene eventually.

Guaiacol 9 is related to the abundant guaiacyl units (Scheme 1) in lignin and, thus, represents a model compound combining hydroxyl and methoxy functionality. It exhibits higher reactivity than catechol 5,^10^ which can be rationalized by rapid demethylation (Scheme 2), which leads to its rapid conversion to catechol 5 over the Brønsted acid sites of the zeolite. Starting from 5, three reactions can proceed: dehydration and decarbonylation to cyclopentadienone 8, dehydroxylation to phenol 1, or dehydration to fulvenone 6.^17–22^ By tracing reactive intermediates utilizing *operando* PEPICO, these reaction pathways were revealed to drive the reaction mechanism of guaiacol catalytic pyrolysis (Scheme 2). Due to the abundance of surface methyl species (SMS), methylation reactions abound and yield methylcyclopentadienes 10, the precursors of fulvene 11 and benzene 3. Secondary chemistry, such as methylation reactions, is also responsible for the formation of cresols 12 and the decarbonylation to yield cylopentadiene 2. By changing the MFI framework to faujasites (FAU), these secondary reactions could be suppressed in guaiacol catalytic pyrolysis thanks to the larger porosity (5.5 *vs.* 7.4 Å) and the resulting higher diffusivity. In addition, the Si/Al ratio was decreased in FAU, which increased the Brønsted acid site density.^22^ Fulvenone 6 and fulvene 11, were both suppressed when the Brønsted acid site density was increased, while the phenol selectivity increased. This can be rationalized by the simultaneous coordination of the two OH groups of catechol 5 straddling two acid sites, which leads to isolation of the hydroxyl groups. As a consequence, the two OH groups cannot efficiently dehydrate to fulvenone 6 thereby increasing the phenol 1 selectivity.^22^

The complexity of the reaction networks increases as the molecular structure converges to that of native lignin with varied functionalization. There has been little research on multifunctionalized lignin model compounds, such as vanillin, syringol, or eugenol, and the respective reaction pathways are not satisfactory understood.^11,27–29^ Thus, the motivation of the present work is to understand the catalytic pyrolysis chemistry of model compounds with a single aromatic unit and up to three different substituents (Scheme 2b). These compounds are representative of the lignin moieties after breaking the linkers, *i.e.*, the α–β, β-O-4, β–β or 4-O-5 bonds. For instance, the guaiacyl unit (G in Scheme 1) may yield vanillin or eugenol through hydrogen transfer at the radical sites after β-O-4 or α–β bond cleavage, whereas syringyl units (S in Scheme 1) may be directly transformed into syringol during lignin depolymerization (Scheme 1). Moreover, our study elucidates the influence of methoxy, carbonyl, and allyl substitution on the reactivity and product distribution (Scheme 2b) of the guaiacol group, completing our results on single-ring aromatic lignin model compound mechanisms over HZSM-5 zeolite.^20–22^ In addition, Kawamoto *et al.* postulated that especially C_3_-units give rise to reactive quinone methide intermediates, which are responsible for the condensation of two ring species *via* rearomatization.^30–32^ Until now, these intermediates could only be indirectly observed based on isolation and characterization of the dimers or by using mass spectrometric methods, which makes it difficult to reveal all isomers of a certain composition.^29^ Our study provides first spectroscopic evidence for three quinone methide derivatives in lignin catalytic pyrolysis. Lastly, this work will contribute to bringing the bottom-up (model compound pyrolysis) and top-down (lignin pyrolysis) strategies closer together to provide a more complete picture of lignin valorization chemistry.^33^

## 2. Results and discussion

The catalytic pyrolysis of the model compounds was carried out at the VUV beamline of the Swiss Light Source using *operando* PEPICO spectroscopy.^19–22^ This technique enables the detection of reactive intermediates and stable products by combining mass spectrometry and photoion mass-selected threshold photoelectron spectroscopy, which is an isomer-selective tool readily applicable to the analysis of reactive flows in, *e.g.*, heterogeneous catalysis.^33–35^

### 2.1 Carbonyl substitution: vanillin

**Fig. 1:**
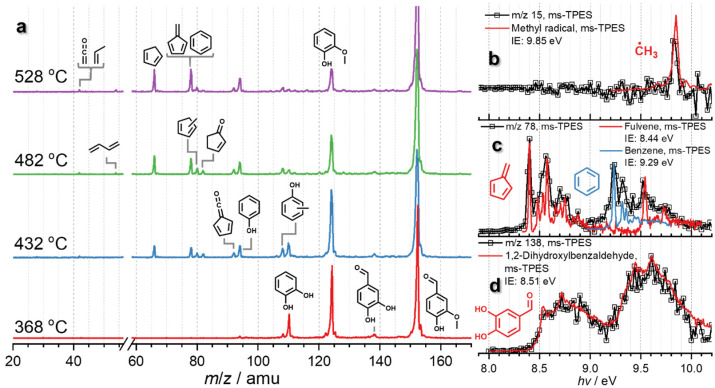
Mass and photoion mass-selected photoelectron spectra of the catalytic fast pyrolysis products of vanillin over HZSM-5. (a) ToF MS recorded at 10.5 eV for 2 min. Reaction conditions: 0.05% vanillin (*m*/*z* 152); HZSM-5 (Si/Al = 25); ∼0.3 bar; 20 sccm Ar. (b–d), ms-TPES of the desorbed methyl radicals (*m*/*z* 15), fulvene (*m*/*z* 78), benzene (*m*/*z* 78), and 1,2-dihydroxybenzaldehyde (*m*/*z* 138), respectively, shown together with Franck–Condon (FC) simulations based on G4 calculations and experimental reference spectra.

Vanillin 13 is a major product in lignin (catalytic) pyrolysis,^36–38^ produced by C–C (α–β) bond cleavage leading to an aldehyde functional group after stabilization of the radical sites (Scheme 1). Fig. 1 shows temperature-dependent mass spectra of vanillin pyrolysis over HZSM-5 (Si/Al = 25), as well as photoion mass-selected threshold photoelectron spectra (ms-TPES), which identified methyl radicals (*m*/*z* 15), fulvene (*m*/*z* 78), benzene (*m*/*z* 78), and 1,2-dihydroxybenzaldehyde (*m*/*z* 138). A detailed speciation *via* reference and Franck–Condon spectral modelling is presented in Fig. S1 in the ESI.† Starting at 368 °C, vanillin (*m*/*z* 152, 13) decomposes to guaiacol (*m*/*z* 124, 9) over HZSM-5, which is the dominant product in the mass spectrum while 1,2-dihydroxybenzaldehyde (*m*/*z* 138, 14), the direct demethylation product of vanillin 13, is barely observed. In addition, we do not find any other aldehyde bearing species, confirming decarbonylation as the first step in the decomposition of vanillin 13. At 432 and 482 °C, guaiacol 9 and catechol 5 decompose further to yield phenol (*m*/*z* 94, 1), benzene (*m*/*z* 78, 3), fulvene (*m*/*z* 78, 11), and cyclopentadiene (*m*/*z* 66, 2). Up to a temperature of 528 °C, *m*/*z* 78 (benzene and fulvene) and cyclopentadiene (*m*/*z* 66) dominate the mass spectrum accompanied by small amounts of ketene and propene (*m*/*z* 42).

Since vanillin 13 readily decarbonylates to guaiacol, reaction pathways from there correspond to the guaiacol decomposition pathways as outlined in Scheme 2. However, the question arises if the aldehyde group leaves as a formyl radical (O=C–H) as proposed by Vasiliou *et al.* or as carbon monoxide.^39^ In the presence of zeolites, formyl radicals are prone to catalytic stabilization by hydrogen transfer to formaldehyde, itself a reactive intermediate with a central role in the zeolite-catalyzed methanol to hydrocarbons process chemistry.^40^ CO and HCHO signals were traced in vanillin catalytic pyrolysis as a function of the temperature (Fig. S2a†). Only the CO signal changes with temperature between 416 and 560 °C, while the formaldehyde mass spectral signal stays constant. However, CO may not be solely produced during vanillin 13 decarbonylation, but also during catechol 5 and guaiacol 9 decomposition, as observed previously.^10,21^ To exclude these CO sources, the reactor temperature was lowered to allow only for the decarbonylation of the aldehyde group. Even at the low temperature of 368 °C, the detected CO signal exceeds the blank signal significantly. In addition, we also investigated benzaldehyde pyrolysis over the same catalyst at a similar temperature to follow the fate of CO and benzene (Fig. S2b†), which also confirmed that the aldehyde group decomposes to CO rather than yielding HCHO.

**Scheme 3:**
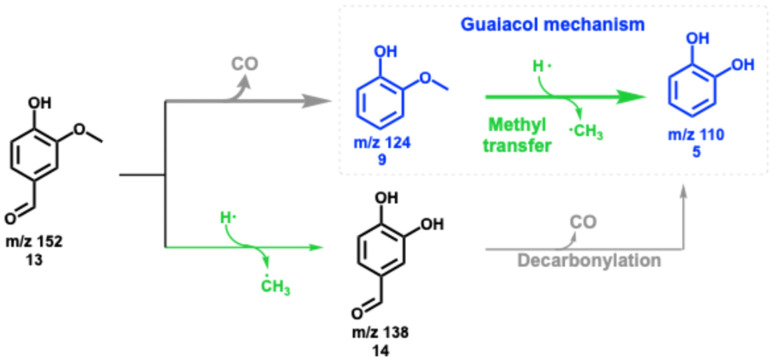
The initial reaction pathways of vanillin 13 in catalytic pyrolysis. Bold arrows represent the dominant reaction pathways, leading to the most abundant species, guaiacol 9 and catechol 5, which react further according to Scheme 2.

In addition to decarbonylation to guaiacol, (9, Scheme 3), vanillin 13 may also be demethylated in a minor channel to 1,2-dihydroxybenzaldehyde 14 in catalytic pyrolysis, yielding traces of signal at *m*/*z* 138, which is readily decarbonylated to catechol. The primary product guaiacol 9 readily demethylates forming catechol 5 and follows the chemistry presented in Scheme 2. Therefore, ketene 6, cyclopentadiene 2, and fulvene 11 formation dominates, while the selectivity towards phenol 1 is low over HZSM-5. However, as we previously described for guaiacol, the mechanism can be fine-tuned by changing the MFI (HZSM-5) to the FAU framework and lowering the Si/Al ratio at the same time. This increases the Brønsted acid site density in FAU and suppresses the formation of fulvenone 6 and fulvene 11, resulting in enhanced phenol 1 selectivity, as shown in Fig. S3.† ^22^ A detailed discussion of this experiment including catalyst characterization can be found in the ESI,† which, together with the guaiacol investigations^21,22^ completes the catalytic pyrolysis reaction mechanism of vanillin over zeolites.

### 2.2 Methoxy substitution: syringol

**Fig. 2:**
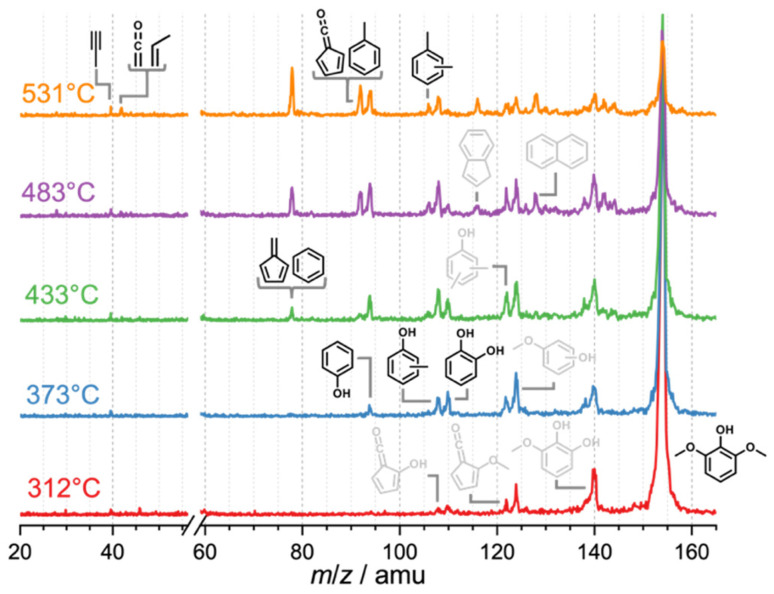
Catalytic pyrolysis of syringol. Temperature-dependent mass spectra recorded at *hν* = 10.5 eV upon syringol catalytic pyrolysis. Grey refers to potential products that could not be unambiguously identified. Reaction conditions: <0.1% syringol (*m*/*z* 154); H-ZSM-5 (Si/Al = 25); <0.5 bar; 20 sccm Ar.

**Scheme 4:**
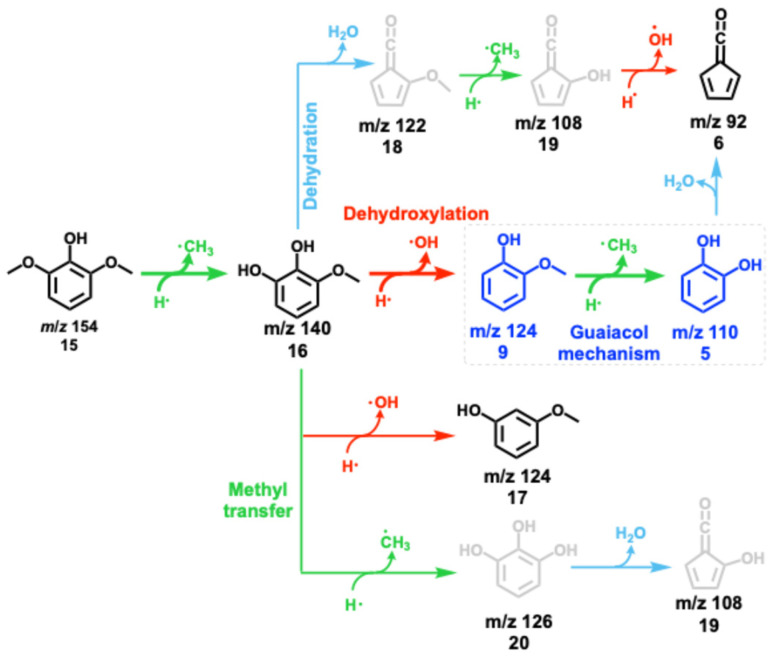
Reaction mechanism of syringol 15 CFP over HZSM-5. Black structures have been detected, while grey ones remain tentatively assigned. Bold arrows represent dominant reaction pathways, leading to the most abundant products. After demethylation and dehydroxylation, syringol CFP chemistry follows that of guaiacol 9.

Syringyl units are one of the three major moieties in the lignin structure and amount to up to 70% in hardwood and herbaceous biomass.^41,42^ These units are converted to syringol and its derivatives during initial lignin cracking and subsequent stabilization *via* hydrogen transfer.^36,43^ Syringol catalytic pyrolysis mass spectra (Fig. 2) as a function of the reactor temperature show intermediates and products of further conversion, which can then be conclusively identified by ms-TPES and photoionization (PI) spectra (Fig. S4 in the ESI†). Syringol 15 decomposition starts at approx. 312 °C, yielding 3-methoxycatechol (*m*/*z* 140, 16), *o*- (9) and *m*-guaiacol (*m*/*z* 124, 17), dimethylphenols (*m*/*z* 122), and cresols (*m*/*z* 108). The HZSM-5 catalyst plays a central role in producing these species, because no products are observed in blank syringol pyrolysis experiment at temperatures up to 531 °C (Fig. S5†). 3-Methoxycatechol (*m*/*z* 140, 16) is identified as the first demethylation product of syringol 15, preferentially dehydroxylating to *o*- and *m*-guaiacol (*m*/*z* 124, Scheme 4), which are both observed in the PI spectrum (Fig. S4†). Note that the S/N ratio was not high enough to obtain the corresponding ms-TPES, but even if it had been, in the absence of vibrational fine structure, the isomer-selective identification of methoxyphenols would be challenging based on their ground-state band in the TPES. At higher reactor temperatures, catechol (*m*/*z* 110, 5), cresols, and xylenes can be identified based on their PI curve (Fig. S4†). It is, thus, unsurprising that typical guaiacol CFP products, such as phenol (*m*/*z* 94, 1), fulvenone (*m*/*z* 92, 6), and toluene (*m*/*z* 92), are also found in the reaction mixture based on their ms-TPES or PI spectrum. By increasing the temperature, the decomposition of 3-methoxycatechol (*m*/*z* 140, 16) as well as of *o*- and *m*-guaiacol (*m*/*z* 124, 9 and 17) are promoted. At 373 °C, catechol (*m*/*z* 110, 5) and phenol (*m*/*z* 94, 1) signals increase. Besides propene (*m*/*z* 42), butadiene (*m*/*z* 44) and cyclopentadiene (*m*/*z* 66, 2), we detect fulvene 11 and benzene (both *m*/*z* 78, 3) as fully deoxygenated hydrocarbons. Upon increasing the temperature, yields of toluene and fulvenone (*m*/*z* 92), benzene and fulvene (*m*/*z* 78), cyclopentadiene (*m*/*z* 66, Fig. S4†), 1,3-butadiene (*m*/*z* 54, Fig. S4†), ketene and propene (*m*/*z* 42), and propyne (*m*/*z* 40) increase gradually. Indene (*m*/*z* 116) and naphthalene (*m*/*z* 128) appear at elevated temperatures contributing to the hydrocarbon pool as coke precursors. Similar decomposition pathways are found during catalytic pyrolysis of benzenediols (5) and methoxyphenols (guaiacol, 9), however, with higher yields of cyclopentadiene (C_5_H_6_, 2) as compared to syringol decomposition. 5 and 9 follow mostly the chemistry in Scheme 2.^10,21^

### 2.3 Propenyl substitution: eugenol

β-O-4 is an abundant linkage between aromatic units in lignin,^7^ and it decomposes to various substituents, such as propyl, allyl, and aldehyde functionalities.^44–46^ In particular, the cleavage of the C–O bond at the β-carbon gives rise to a hydroxylated C_3_ group, which can eliminate water to afford eugenol, one of the main thermal treatment products of lignin.^46–49^

**Fig. 3:**
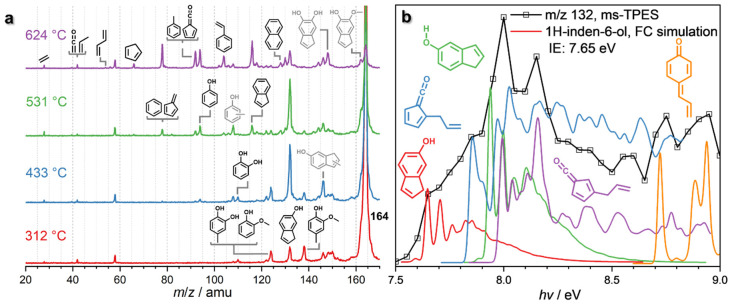
(a) ToF MS recorded at *hν* = 10.5 eV upon eugenol catalytic pyrolysis using HZSM-5. Grey structures denote potential or inconclusive assignments. Reaction conditions: <0.5% eugenol (*m*/*z* 164); HZSM-5 (Si/Al = 25); <0.5 bar; 20 sccm Ar. (b) The ms-TPES of *m*/*z* 132 evidences the formation of indenols and the quinone methide derivative, 4-allylidenecyclohexa-2,5-dien-1-one.

Eugenol (*m*/*z* 164) already decomposes at 312 °C (Fig. 3a and Fig. S6†) over HZSM-5, yielding intermediates and products at *m*/*z* 150, 146, 138, 132, and 124, which are assigned to 4-methylguaiacol (*m*/*z* 138), 1*H*-inden-6-ol (*m*/*z* 132) and isomers, guaiacol and 4-methylcatechol (both *m*/*z* 124) (Fig. S6†). At 433 °C, *m*/*z* 132 (1*H*-inden-6-ol and isomers) dominates the mass spectrum, accompanied by a signal *m*/*z* 146, which is probably a methylation product of 1*H*-inden-6-ol or its isomers. However, due to the low S/N of this peak, we could not fully confirm the assignment. Additional routes forming allyl-substituted fulvenone ketenes, which were also suggested by Zhou *et al.* are possible, but the features cannot be clearly identified, due to spectral congestion (Fig. 3b).^29^ A more definitive assignment can be provided for the substituted quinone methide at *m*/*z* 132 (Fig. S6†). The two features above 8.8 eV photon energy agree well with the FC simulation of this reactive intermediate, tentatively responsible for the condensation of lignin monomers according to Kotake *et al.* (*vide infra*).^30–32^

In addition, lighter products, such as catechol (*m*/*z* 110) and phenol (*m*/*z* 94), are produced within the same temperature window. At 531 °C, these species increase together with indene (*m*/*z* 116), while methylated 1*H*-inden-6-ol isomers (*m*/*z* 146) decrease. When the reactor temperature reaches 624 °C, *m*/*z* 132 declines and indene, phenol, *m*/*z* 92 (toluene and fulvenone), and *m*/*z* 78 (benzene and fulvene) are the main products, accompanied by cyclopentadiene (*m*/*z* 66), 1,3-butadiene (*m*/*z* 54), as well as propene and ketene (*m*/*z* 42). At high temperatures, *m*/*z* 162 is also observed, likely produced by cyclization and dehydrogenation of eugenol. The detection of *m*/*z* 162 at low temperatures is difficult, though, because of the dominant eugenol signal at *m*/*z* 164 (Fig. 3a).

**Fig. 4:**
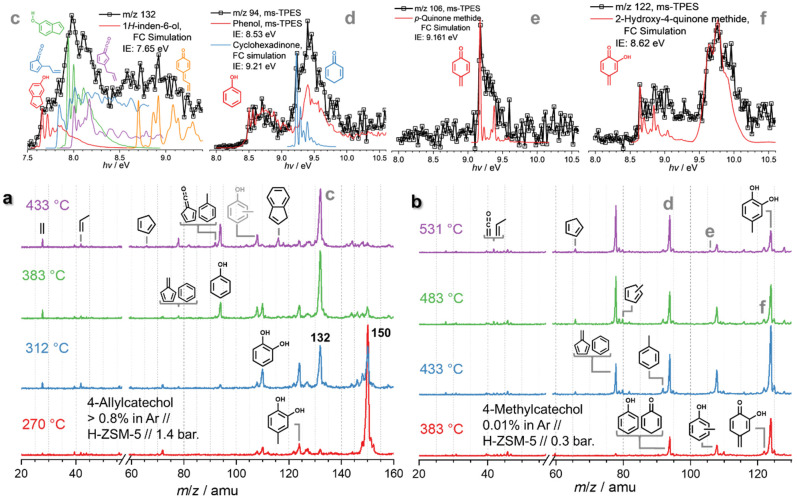
*Operando* mass spectra in catalytic pyrolysis of (a) 4-allylcatechol and (b) 4-methylcatechol over HZSM-5. (c–f) ms-TPES of *m*/*z* 132 (from a), 94, 106, and 122 (from b), respectively.

Due to the complexity of the chemistry, involving parallel demethylation and cyclization pathways as well as of quinone methide intermediates, the eugenol reaction mechanism needs to be further refined by studying simplified model compounds, namely allylbenzene, 4-allylcatechol, and 4-methylcatechol, to understand the behaviour and interactions of the individual functional groups (Fig. 4).

#### 2.3.1 Understanding the reactivity of C_3_ units: allylbenzene

**Scheme 5:**
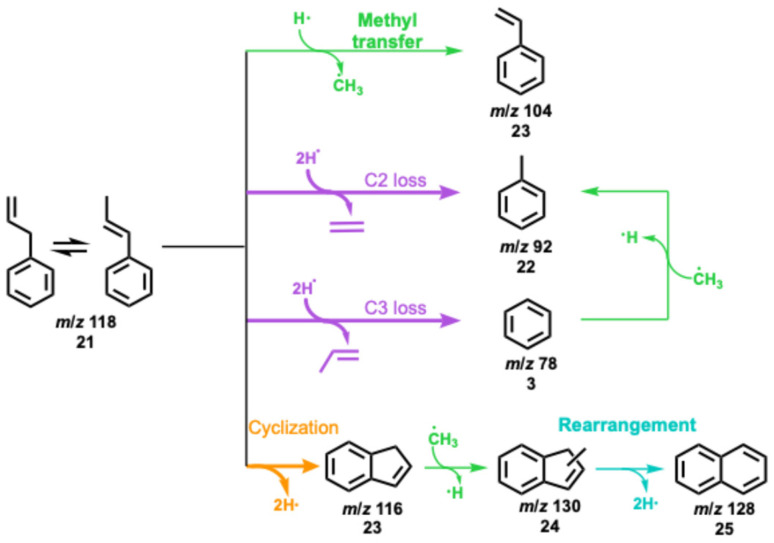
Reaction pathways observed during allylbenzene CFP. Thick lines indicate dominant product formation channels.

Based on the observation of indene, indanol, and dihydroxyindene in eugenol catalytic pyrolysis, a new ring closure pathway arises besides ring condensation in the hydrocarbon pool mechanism leading to, *e.g.*, naphthalene from guaiacol. To isolate the chemistry of C_3_ ligands, we analysed the decomposition channels of allylbenzene over HZSM-5 *via* mass spectrometry and ms-TPE spectroscopy (Fig. S7 and S8†). Based on the observed intermediates and products, Scheme 5 summarizes the reaction pathways of allylbenzene 21 over HZSM-5. The removal of propylene (C_3_-loss) to yield benzene (*m*/*z* 78, 3) is the reaction with the highest selectivity. Alternatively, allylbenzene may also decompose to toluene (*m*/*z* 92, 22, C_2_-loss) and styrene (*m*/*z* 104, 23, C_1_-loss), giving rise to ethylene (*m*/*z* 28) and surface methyl species. The resultant SMS may in turn methylate benzene 3 or indene 23 to produce toluene 22 or naphthalene 25, respectively, after rearrangement. Beside these reaction pathways, ring closure over HZSM-5 is followed readily by dehydrogenation yielding indene 23, which explains the high indene 23 abundance in the product distribution of eugenol catalytic pyrolysis at 624 °C (Fig. 3). In addition, indene 23 can also react with SMS to form naphthalene 25*via* methyl indene (*m*/*z* 130, 24). This finding suggests that the allyl functional groups, as produced in β-O-4 cleavage, are important contributors to coke. The observation of 1-phenylpropene 21 as an allylbenzene isomer (Fig. S8†), confirms rapid isomerization (equilibration) of the double bond in the system. In conclusion, allylbenzene 21 exhibits high reactivity over HZSM-5 thanks to the allyl group, which can participate in multiple reactions already in mild conditions leading to rapid branching. Most importantly, ring closure in allyl arenes forms indene analogues, which is followed by production of polycyclic aromatic hydrocarbons, contributing to coke inception.

#### 2.3.2 Supressing methylation: 4-allylcatechol

As ring closure and C_3_ loss could selectively be observed in allylbenzene 21 (Scheme 5), it is likely that they also happen in eugenol CFP and compete with demethylation at the methoxy site followed by hydroxyl formation at low temperatures over HZSM-5.^21,22^ However, abundant surface methyl species convolute the chemistry and lead to the formation of cresol and methyl cyclopentadienes. Thus, by suppressing methylation reactions, the chemistry can be simplified. This makes 4-allylcatechol 26 an ideal model compound, since it is also a reaction product of eugenol CFP, contributing to the *m*/*z* 150 signal (Fig. 4a). Temperature-dependent mass spectra of 4-allylcatechol over HZSM-5 (Fig. 4a) show an initiation of the reaction already at 270 °C to yield *m*/*z* 132, 124, and 110. When the temperature is increased to 312 °C, *m*/*z* 132 dominates the mass spectrum like in eugenol (Fig. 3). Blank experiments show no product formation below 531 °C (Fig. S9†) proving that the *m*/*z* 132, 124, and 110 peaks are due to catalytic pyrolysis. The ms-TPES of *m*/*z* 132 (Fig. S10†) compares well with the one taken during eugenol CFP (Fig. S6†) and can be assigned to hydroxyindene isomers. Because of them being almost isoenergetic (Fig. S11†), we cannot rule any of them out from a thermochemical point of view. Nevertheless, 1*H*-inden-6-ol sticks out among the isomers due to its low ionization energy and a vibrational structure matching the experimental result well. A substituted fulvenone at *m*/*z* 132 may also contribute to the feature at 7.85 eV, but fulvenones lie *ca.* 0.05–0.07 eV (5–7 kJ mol^−1^) higher than the most stable hydroxyindenes (Fig. S11†). Vinyl substituted quinone methide, 4-allylidenecyclohexa-2,5-dien-1-one, is slightly lower in energy, has a calculated ionization energy of 8.77 eV (G4) and its FC simulation shows excellent agreement with the experimental ms-TPES (see Fig. S10,†*m*/*z* 132). The quinone methide features are also found in the eugenol CFP *m*/*z* 132 ms-TPES (Fig. S6,†Fig. 3b), further confirming the formation of this quinone methide derivative.

**Scheme 6:**
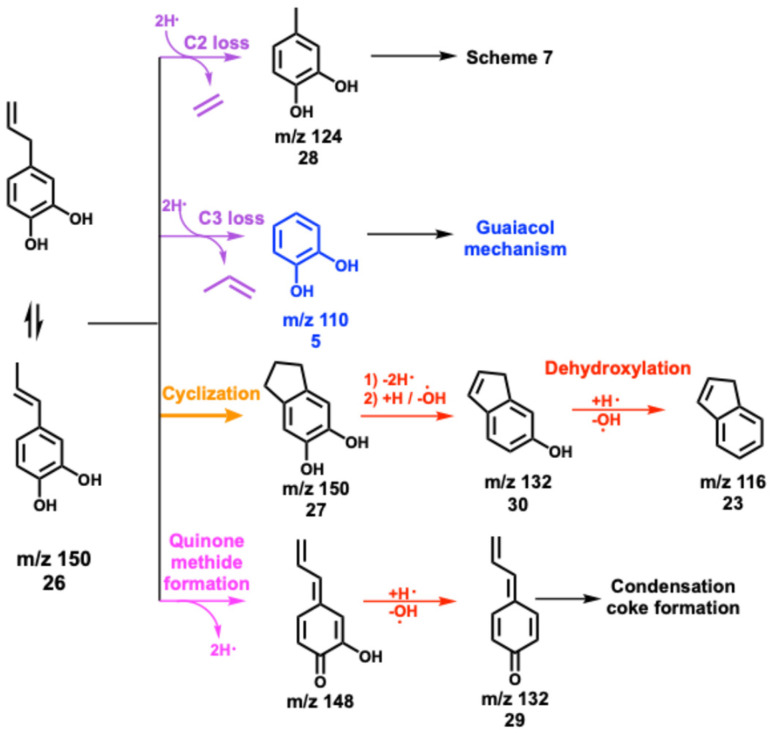
Reaction pathways of 4-allylcatechol over HZSM-5.

Besides, *m*/*z* 124 and 110 formation are promoted at 312 °C and the peaks are assigned to 4-methylcatechol and catechol, respectively (Fig. S10†). When the temperature increases to 383 °C, *m*/*z* 150 decomposes mostly and *m*/*z* 132 dominates the mass spectra. Besides the catechol derivatives, smaller products, such as cresols (*m*/*z* 108), phenol (*m*/*z* 94), as well as fulvene and benzene (*m*/*z* 78) are formed. When heating to 433 °C, fulvenone and toluene (*m*/*z* 92), as well as benzene and fulvene (*m*/*z* 78) are observed, which originate from the decomposition of 4-methylcatechol and catechol. Meanwhile, indene appears, which is formed by dehydroxylation of hydroxyindene (*m*/*z* 132), similar to the phenol to benzene chemistry.^10^ Notably, the ms-TPES of *m*/*z* 150 (Fig. S10†) does not agree with the pure 4-allyl benzenediol spectrum recorded at room temperature. However, the onset of the ms-TPES is reproduced by the FC simulations of two dihydroxyindane isomers (*m*/*z* 150, Fig. S10†), confirming the propensity of the allyl group towards ring closure. The products observed in eugenol also appear in 4-allylcatechol CFP, albeit with a significant reduction of the methylation reaction. The CFP chemistry of 4-allylcatechol 26 is summarized in Scheme 6.

Due to the rapid isomerization of the allyl group, 4-allylcatechol 26, 4-(1-propenyl)-catechol and dihydroxyindane isomers coexist (*m*/*z* 150, Scheme 6), which is also seen during allylbenzene CFP (see Fig. S7 and S8†). Ethylene and propylene loss produces 4-methylcatechol (*m*/*z* 124, 28) and catechol (*m*/*z* 110, 5), respectively. Guaiacol and catechol 5 follow the chemistry as shown in Scheme 2.^22^ Because of the two hydroxyl groups in 27 (Scheme 6), dehydroxylation takes place, giving rise to 1*H*-inden-6-ol (30). A fourth 4-allylcatechol CFP channel gives rise to 3-hydroxy-4-allyl-cyclohexadienone (*m*/*z* 148) following a hydrogen transfer, followed by rapid dehydroxylation to 4-allyl-cyclohexadienone (29, Scheme 6), *i.e.*, a substituted quinone methide species, also proposed by Zhou *et al.* along with fulvenone isomers in a mass spectrometry study of eugenol pyrolysis.^29^ Kawamoto postulated that this quinone methide intermediate is largely responsible for the condensation of lignin monomer units and is, thus, a prime contributor to coke formation.^30–32^

Compared to allylbenzene, 4-allylcatechol 26 shows a more complex reaction mechanism thanks to the presence of two hydroxyl groups. Dehydroxylation and dehydration compete with reactions centred at the allyl site. This is also reflected by the formation of 1*H*-inden-6-ol and quinone methide (both *m*/*z* 132), which dominate the product distribution at moderate temperatures, nicely mirroring the eugenol results and thus revealing a subset of the eugenol CFP chemistry.

#### 2.3.3 Supressing ring closure: 4-methylcatechol

The allylbenzene and 4-allylcatechol analogues allowed us to isolate the C_3_ chemistry and suppress surface methyl species in the eugenol CFP mechanism. Ring closure is always dominant in the presence of the allyl group. 4-Methylcatechol is one of the primary products in the eugenol and 4-allylcatechol catalytic pyrolysis and its abundance is similar to the one of *m*/*z* 132 at 312 °C (Fig. 3 and 4a). Selectively supressing ring closure reactions due to the allyl group and revealing the CFP reaction mechanism of this central species will also help us complete the CFP reaction mechanism of eugenol in particular and analogous intermediates in lignin in general. First decomposition products of 4-methylcatechol over HZSM-5 are formed at 383 °C (Fig. 4b and Fig. S12†), in contrast to non-catalytic pyrolysis (Fig. S13†), which only starts at *ca.* 530 °C. Besides the most abundant benzene and fulvene (*m*/*z* 78), phenol (*m*/*z* 94) as well as cresol (*m*/*z* 108) peaks in the mass spectra, we identify cyclopentadiene (*m*/*z* 66), methyl-cyclopentadienes (*m*/*z* 80), and toluene (*m*/*z* 92) in the reaction mixture. The early intermediates and products are 2-hydroxy-4-quinone methide (*m*/*z* 122), cresols (*m*/*z* 108), phenol (*m*/*z* 94) and cyclohexa-2,5-dien-1-one (*m*/*z* 94) at 383 °C. 2-Hydroxy-4-quinone methide is a reactive (non-aromatic) intermediate produced by dehydrogenation of 4-methylcatechol, aligning well with the 4-allylidenecyclohexa-2,5-dien-1-one (29) formation discussed above. Notably, *semi*-quinone generation is analogous to *para*-quinone production during hydroquinone (1,4-benzenediol) CFP over HZSM-5 *via* dehydrogenation.^10^ Decarbonylation of (semi-) quinones gives rise to *m*/*z* 94, which we identify as phenol and cyclohexa-2,5-dien-1-one by its ms-TPES (see Fig. S12†). The peak at *m*/*z* 108 is due to cresols, and increases with the reactor temperature at the beginning. However, cresols decompose further at high temperatures towards lighter species, in line with previous findings.^21^ Dehydrogenation to afford the reactive *para*-quinone methide at *m*/*z* 106 can be identified as a potential decomposition channel, according to the ms-TPES (see Fig. S12†). Based on these observations, we can summarize the mechanism of 4-methylcatechol as follows.

**Scheme 7:**
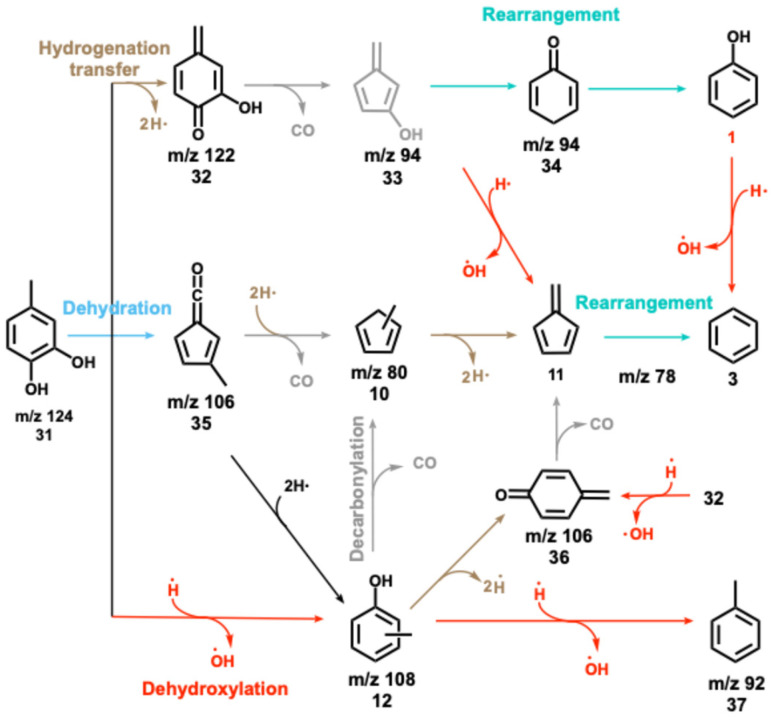
CFP mechanism of 4-methylcatechol.

The 4-methylcatechol 31 pyrolysis is initiated by dehydrogenation to yield 2-hydroxy-4-quinone methide (*m*/*z* 122, 32), which rapidly decarbonylates to *m*/*z* 94 isomers 33, yielding phenol as a one of the most abundant products (Scheme 7). The observation of cyclohexa-2,5-dien-1-one (34, also *m*/*z* 94) as intermediate on the way to yield phenol hints at a complex reaction mechanism, likely involving multiple rearrangement pathways, with an intermediately formed hydroxyfulvene (33, *m*/*z* 94), which could not be identified directly. Benzene 3 and fulvene 11 are the most abundant carriers of the mass signal at *m*/*z* 78. While the former can be formed *via* dehydroxylation of phenol (1 → 3, Scheme 7),^10^ fulvene 11 must originate from a different CFP channel, which may proceed *via* methylfulvenone 35, similar to the catechol dehydration reaction (5 → 1, Scheme 2).^10^ The methylfulvenone ketene 35 may decarbonylate giving rise to methylcyclopentadienyl (35 → 10, *m*/*z* 80) which is known to dehydrogenate to fulvene 11.^20,23^ Alternatively, 35 may also hydrogenate and ring-expand to yield cresols 12, as observed in our reaction mixture. The decreasing cresol 12 selectivity at higher temperatures points to their decomposition, likely *via* dehydrogenation yielding semi-quinone 36, observed in the ms-TPES at *m*/*z* 106. In addition, 36 can also be formed *via* a direct dehydroxylation of its hydroxy derivative (32 → 36). Like in *ortho*- and *para*-quinones, decarbonylation also occurs in *semi*-quinones, which explains the formation of fulvene (36 → 11). Methylcyclopentadienes 10 mark a second route to fulvene 11, while cyclopentadiene 2 is formed in phenol 1 decarbonylation (Scheme 2).^10,18^ Similar to catechol, dehydroxylation is one of the initial decomposition steps, giving rise to cresols (31 → 12, *m*/*z* 108, Scheme 7) and toluene (12 → 37). On the one hand, dehydration of the vicinal hydroxyl groups is likely to give rise to methylfulvenone 35, which could, however, not be directly observed. On the other hand, the appearance of methylcyclopentadienes 10 and cresols 12 is proof for the existence of this channel.^10^

#### 2.3.4 Eugenol catalytic pyrolysis mechanism

**Scheme 8:**
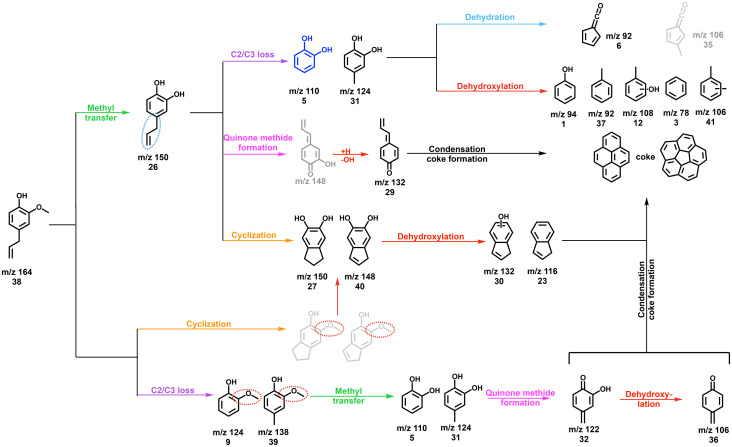
The main initial decomposition pathways of eugenol in HZSM-5-catalyzed pyrolysis.

Based on the intermediates and products detected in the CFP of eugenol, allylbenzene, 4-allylcatechol, and 4-methylcatechol, the main reaction pathways and products of eugenol 38 upon catalytic pyrolysis can now be summarized (Scheme 8). The reactivity of the methoxy and allyl groups is the highest. Methoxy groups dominantly demethylate resulting in 4-allylcatechol 26 formation, while the allyl group can decompose *via* C_2_ or C_3_ loss yielding guaiacol 9 and derivatives 39 or cyclize to indanes (27), indenes (40), and their derivatives, providing viable pathways to the most abundant intermediate at *m*/*z* 132 (30). Guaiacol 9, 4-allylcatechol 26, and their derivatives (39) decompose to catechol 5 and its derivatives 31, which dehydroxylate to phenol 1, benzene 3, and derivatives (37, 12, and 41), or dehydrate to fulvenone 6 and similar structures 35. Hydroxylated species are prone to dehydroxylation, giving rise to benzene 1, xylenes 41, cresols 12, phenol 1, and indene 23. Quinone methides are an important intermediate formed *via* hydrogen transfer and dehydroxylation (26 → 29) or *via* 4-methyl catechol (31 → 32 → 36).

In general, the comparable reactivity of allyl and methoxy groups results in multiple active pathways in parallel. For example, demethylation of the methoxy group may occur as the allyl group participates in ring closure, resulting in uncontrollable reactions. The allyl group plays a major role in branching the reaction pathways *via* allyl fragmentation. Moreover, the allyl group can cyclize leading to indene formation, a potent coke precursor.

## 3. Summary & discussion

**Scheme 9:**
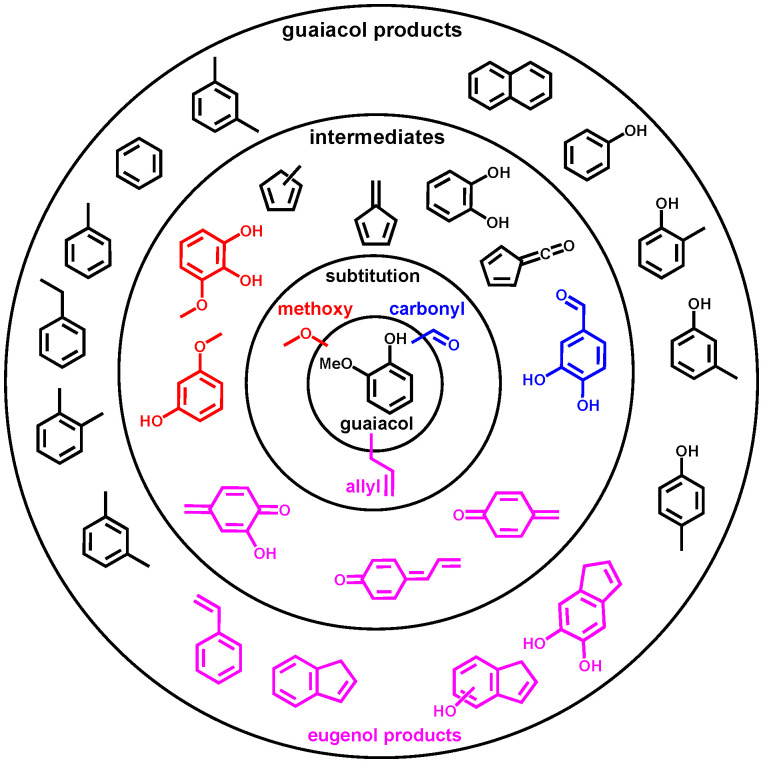
Summary of the intermediates and product distributions in three substituted guaiacols (black): specific reactivities for vanillin (blue), eugenol (pink) and syringol (red) are colour coded.

The catalytic pyrolysis pathways of substituted guaiacol model compounds, syringol (methoxy), vanillin (carbonyl), and eugenol (allyl) were investigated over HZSM-5, and reaction pathways are proposed based on the detection of stable and reactive intermediates. Scheme 9 summarizes the changes in products and intermediates in the CFP of substituted guaiacols. Black are guaiacol-related products observed in all three functionalized guaiacols, while the channels unique to vanillin, eugenol and syringol are blue, pink, and red respectively. Vanillin decarbonylation to guaiacol is the predominant channel at the first decomposition stage. The aldehyde group has a high reactivity and is distinctly converted to CO in the initial reaction, rather than to formaldehyde. As a minor channel, vanillin demethylates to 1,2-dihydroxybenzaldehyde (Scheme 9, blue), which, together with guaiacol, continues to decompose to catechol. Therefore, the vanillin reaction mechanism (Scheme 3) overlaps almost completely with that of guaiacol (Scheme 2). This offers an avenue to increase the phenol selectivities in vanillin CFP by utilizing faujasites and changing the Si/Al ratio and thereby the Brønsted acid site density. Methoxylated guaiacol, *i.e.*, syringol, primarily demethylates to 3-methoxycatechol (Scheme 5). Further dehydroxylation then yields *o*- and *m*-guaiacol. In minor channels, 3-methoxycatechol (Scheme 9, red) may produce 2-hydroxyfulvenone *via* demethylation of 2-methoxyfulvenone or dehydration of 1,2,5-trihydroxybenzene. A strong propensity towards dehydroxylation and dehydration reactions in syringol leads to a similar product distribution to that of guaiacol (Scheme 2), although less five-membered ring species are formed in syringol. This may be owing to the decreased importance of fulvenone (*c*-C_5_H_4_=C=O) chemistry in syringol compared with vanillin and guaiacol over HZSM-5, as deoxygenation of methoxy and hydroxyl groups in syringol and its demethylation product, 2-methoxycatechol dominate.

Eugenol has a more intricate reaction mechanism than syringol or vanillin, owing to the highly reactive allyl (C_3_H_5_–) group in the *para* position. This initiates additional pyrolysis chemistry at temperatures as low as 250 °C. The allyl group does not only decompose *via* C1/C2/C3 cleavage but also initiates ring closure resulting in the formation of indene derivatives (Scheme 9, pink). This is further confirmed by studying the catalytic pyrolysis of allylbenzene (Scheme 5) and 4-allylcatechol (Scheme 6). These analogues exhibit a simpler mechanism, as methylation reactions are suppressed. Most notably, 4-allylidenecyclohexa-2,5-dien-1-one, a reactive quinone methide (Scheme 9, pink), has been detected, which is responsible for the condensation lignin monomers, triggering coke formation and the deactivation of the catalyst. Moreover, 4-methylcatechol (Scheme 7), one of main products of 4-allylcatechol and eugenol, is found to be prone to dehydrogenation yielding 2-hydroxy-4-quinone methide. This species yields cyclohexa-2,5-dien-1-one afterwards, a precursor to phenol and fulvene following decarbonylation (Scheme 7). Thereby, a new, alternative reaction pathway is established to phenols besides benzenediol dehydroxylation. Based on these results, we establish the main reaction pathways of eugenol decomposition upon catalytic pyrolysis (Scheme 8). These channels significantly differ from the ones dominating in guaiacol, syringol, and vanillin, because of the highly reactive allyl group. Consequently, ring closing reactions afford 5-and 6-membered ring species, such as indenes and naphthalenes. This shows that the complicated and in part novel CFP reaction mechanism of eugenol is revealed thanks to the isomer-selectivity of *operando* photoelectron photoion coincidence spectroscopy and the judicious choice of model compounds (Scheme 9). Intriguingly, varying the functional groups on the guaiacol framework have convinced us that, in this case, the chemistry is additive, and the functional groups affect it in their own orthogonal way. This contrasts with the observation in the case of the vicinal OHs, which sometimes (at low Brønsted acid site densities) couple.^22^ This means that the mechanism could be reconstructed as a sum of the chemistry of the individual functional groups, depending on their reaction barrier (*e.g.*, CHO leaves first) and reaction paths.

The three investigated guaiacol-based model compounds are ubiquitous monomers in the lignin structure and formed as pyrolysis products. By understanding their chemistry, the elementary reactions are unveiled to yield the primary, secondary, and tertiary products. This aids a mechanism-based optimization of the selectivity. While syringol shows a similar product distribution as guaiacol, vanillin is perfectly on par with the guaiacol chemistry. This means that selectivities in the latter could be controlled by tuning the Si/Al ratio of faujasites.^22^ A low Si/Al ratio increases the selectivity towards phenol, an important platform chemical. On the one hand, if we take lignin with high guaiacyl density or pre-treat lignin to increase the amount vanillin as primary product, the phenol selectivity may be increased, increasing the economic viability of the process. On the other hand, the formation of eugenol during lignin treatment should be avoided as the reactive allyl group is prone to ring closing reactions. This pathway increases the probability that reactive coke precursors, such as quinone methides, indene, and naphthalene, form, which lead to condensation of aromatics to dimers and larger PAHs and eventually coke, blocking the zeolite pores and effectively deactivating the catalyst. Additionally, quinone methides are prone to decarbonylation to fulvene and phenol.

Both vanillin and eugenol can be derived from the β-O-4 dimer structure, and one way to control the selectivity would be to break the C–C bond at the α–β position selectively to produce vanillin, rather than cleaving of the C–O bond and eliminating water to form the allyl groups, as shown in Scheme 1. This shows how, by the detection of new reactive species, such as the semi-quinones, as well as stable reaction products, *operando* PEPICO can aid the process fine-tuning to control product selectivities in a more targeted way in the catalytic fast pyrolysis of lignin.

## Author contributions

ZP: Investigation (lead), formal analysis (lead), writing – original draft (equal), XW: investigation (supporting), formal analysis (supporting). AB: Writing – review & editing (supporting), formal analysis (supporting), JAVB: writing – review & editing (supporting), supervision (supporting), PH: conceptualization (lead), supervision (lead), funding (lead), writing – original draft (equal), formal analysis (supporting).

## Data availability

The data supporting this article have been included in the ESI.† The derived and raw data can be obtained from: https://doi.org/10.16907/1ac46f03-6e1b-4230-b5c0-3df2cec87b5e and https://doi.org/10.5281/zenodo.13377729.

## Conflicts of interest

There are no conflicts to declare.

## Supplementary Material

GC-026-D4GC03143A-s001
