# Supplementary material for: Catalytic pyrolysis mechanism of lignin moieties driven by aldehyde, hydroxyl, methoxy, and allyl functionalization: the role of reactive quinone methide and ketene intermediates
Source: Green Chem. 2024 Aug 30;26(18):9899–910. doi: 10.1039/d4gc03143a (PMC11363027; doi:10.1039/d4gc03143a)
Supplement: GC-026-D4GC03143A-s001 [file GC-026-D4GC03143A-s001.pdf]

Supplementary Information (SI) for Green Chemistry.
This journal is © The Royal Society of Chemistry 2024

Electronic Supplementary Information

Catalytic pyrolysis mechanism of lignin moieties driven by aldehyde, hydroxyl, methoxy, and allyl functionalization: The Role of Reactive Quinone Methide and Ketene Intermediates

*Zeyou Pan[a,b], Xiangkun Wu[a,†], Andras Bodi[a], Jeroen A. van Bokhoven[a,b], Patrick Hemberger[a]**

[a.] *Zeyou Pan, Xiangkun Wu, Andras Bodi, Jeroen A. van Bokhoven and Patrick Hemberger Paul Scherrer Institute, 5232 Villigen, Switzerland. E-mail: patrick.hemberger@psi.ch.*

[b.] *Zeyou Pan, Jeroen A. van Bokhoven Institute for Chemical and Bioengineering, Department of Chemistry and Applied Biosciences, ETH Zurich, 8093 Zurich, Switzerland*

† Current adress: Environment Research Institute, Shandong University, Qingdao 266237, China.

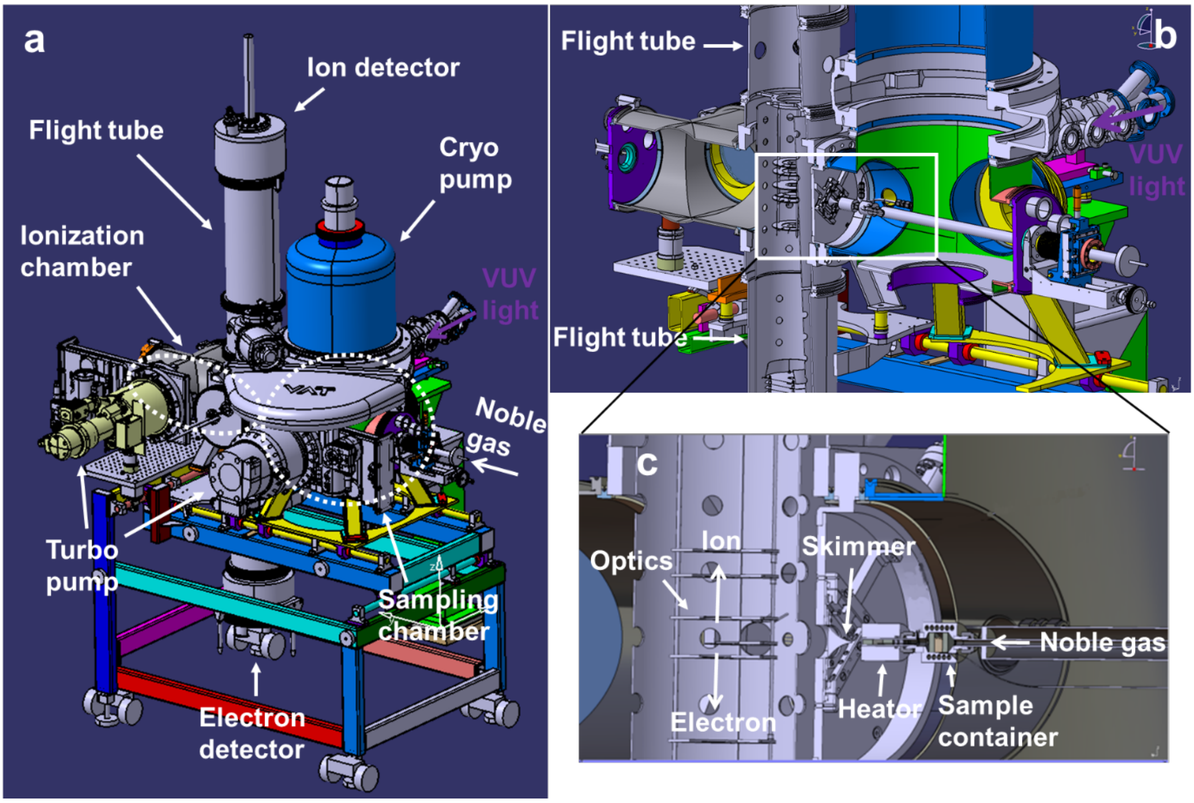

Scheme S1 Catalytic pyrolysis - photoelectron photoion coincidence (PEPICO) setup.

Experimental and Computational Section

Catalytic pyrolysis - photoelectron photoion coincidence (PEPICO) spectroscopy

Catalytic pyrolysis of lignin model compounds is carried out using operando photoelectron photoion coincidence spectroscopy (see **Scheme S1**). The model compounds (vanillin, syringol, eugenol, allylbenzene, 4-allylbenzenediol, and 4-methylcatechol) are vaporized in a heated sample container, diluted in argon and constantly fed into a quartz glass reactor, which holds 20–50 mg of H-ZSM-5 between two quartz wool plugs.[1] The catalyst is heated by a home-built resistively heated oven, and the temperature was measured at the outside of the reactor by a type K thermocouple. After adsorption, reaction and desorption, the mixture of intermediates, products, and reactants leaves the hot reactor. Due to the pressure gap between the reactor (0.1–0.5 bar, abs.) and sampling chamber ($< 10^{-4}$ mbar), a fast, effusive molecular beam is formed, in which molecules travel at nearly the speed of sound, preserving reactive intermediates. Before the molecular beam enters the photoionization chamber, it passes a 2 mm diameter conical skimmer separating the source from the detection chamber. The sample arrives in the ionization region of the detection chamber, is intersected with the vacuum ultraviolet radiation from the VUV beamline at Paul Scherrer Institute,[2] and is ionized, yielding photoions and photoelectrons. Both are accelerated in opposite directions via velocity map imaging optics onto the respective detectors and detected in delayed coincidence. Operando PEPICO techniques offer not only the mass information by time-of-flight mass spectroscopy but also the electronic structure information of the neutral via the photoion mass-selected threshold photoelectron spectrum (ms-TPES).[3,4] Together with reference spectra or Franck–Condon spectral modelling reactive intermediates, products or the reactant can be isomer-selectively be detected.[5] ms-TPES were corrected for false coincidences and the hot electrons were subtracted using the strategy of Sztaray and Baer.[2,6]

Samples

Vanillin (Sigma-Aldrich, 99%), syringol (Sigma-Aldrich, 99%), eugenol (⩾99%), allylbenzene (Sigma-Aldrich, 98%), 4-allylbenzenediol (Fluorochem, 95%), 4-methylcatechol (Sigma-Aldrich, ⩾95%) were used as received.

The ZSM-5 (Si/Al=25), Faujasite (FAU40 (Si/Al=25) and FAU15 (Si/Al=15)) were purchased from Zeolyst International. All zeolites were heated for about 4 h using a heating rate of 2 °C min^{-1}, then calcined in static air at 550 °C for 6 h before use.

Computational details

The Gaussian 16 rev. A.03 and C.01 suite of programs was used to carry out quantum chemical computations. Optimized geometries and vibrational frequencies were utilized in the Franck–Condon (FC) simulations applying density functional theory at the B3LYP/6-31G(d,p) or B3LYP/6-311++G (d,p) level. The stick spectra were convoluted with a Gaussian function and compared to the experimental ms-TPES for isomer-specific assignment.[28,38] The adiabatic ionization energies or relative energies for intermediates and products were calculated using the G4 composite method.[8]

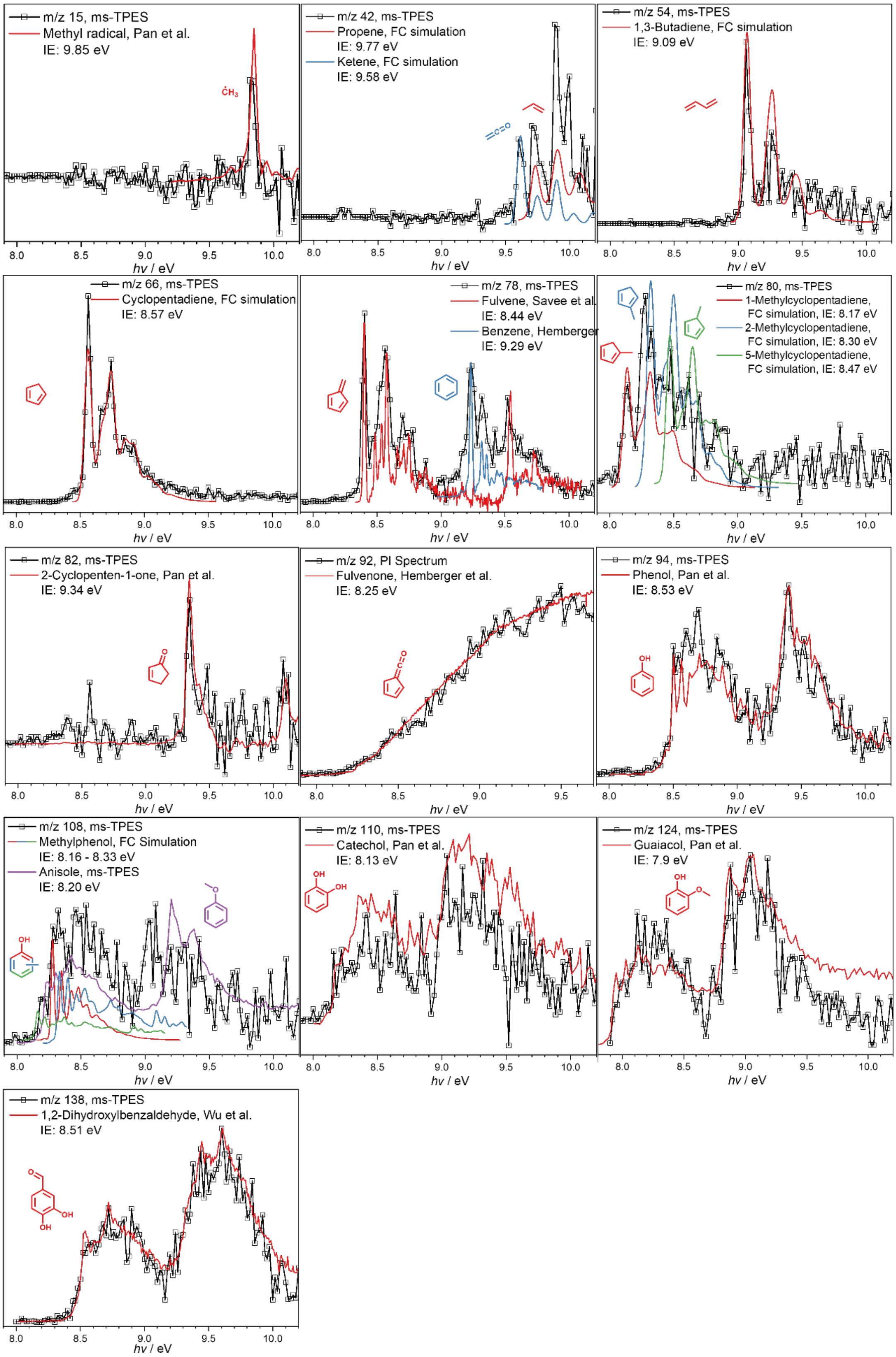

Figure S1 Photoion mass-selected threshold photoelectron spectra (ms-TPES) or photoionization spectra of intermediates and products upon vanillin catalytic pyrolysis, identified based on FC simulations combined with G4 ionization energy (IE) calculations or reference spectra.[9–12]

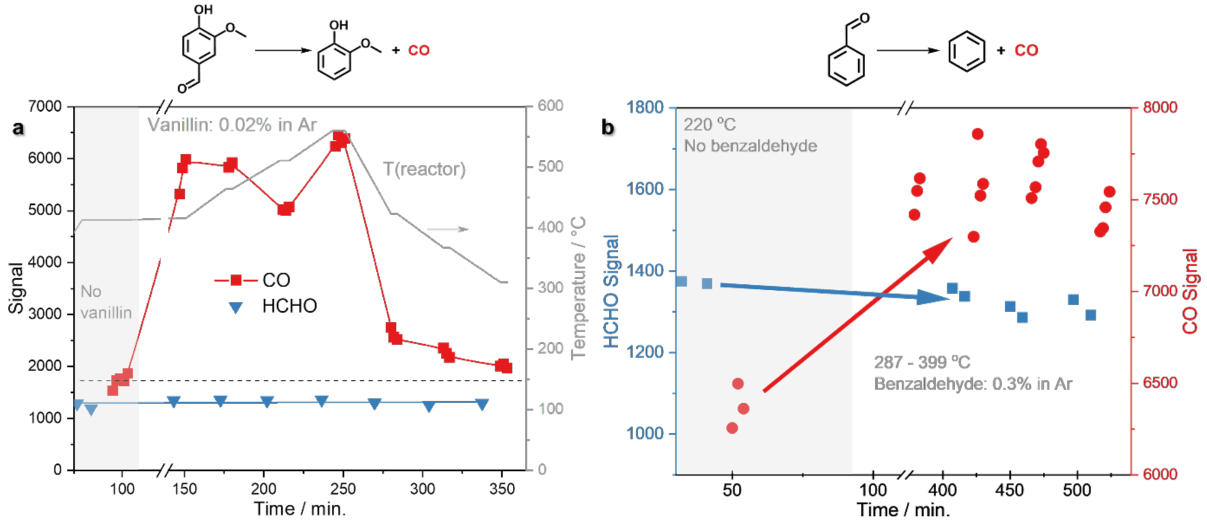

Figure S2 CO and HCHO signals upon catalytic pyrolysis of vanillin and benzaldehyde. **a**) The signals of CO and HCHO in vanillin catalytic pyrolysis using H-ZSM-5 as catalyst. Reaction conditions: Vanillin; H-ZSM-5; 0.3–0.7 bar; 20 sccm Ar. **b**) The signals of CO and HCHO upon benzaldehyde catalytic pyrolysis. Reaction conditions: Benzaldehyde; 220–399 °C; H-ZSM-5; ~ 0.6 bar; 20 sccm in total.

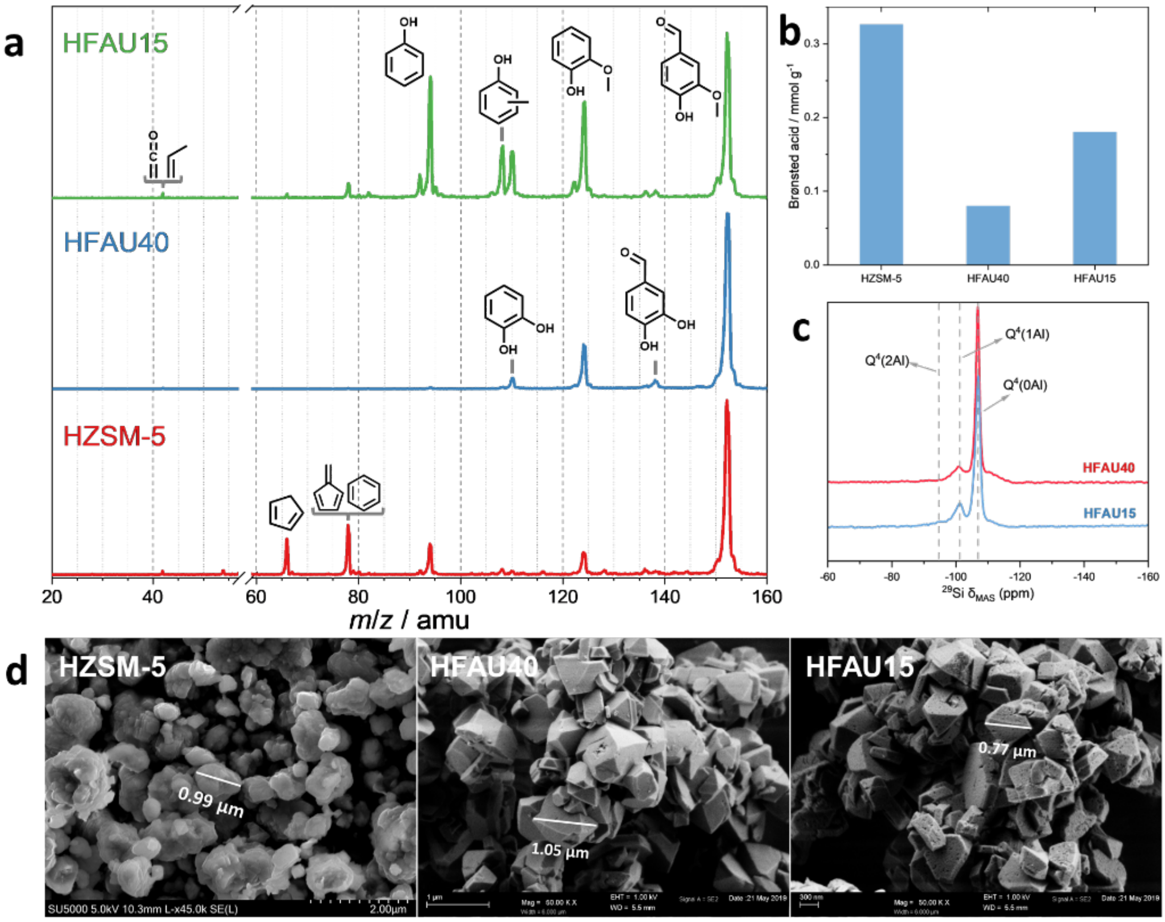

e

Catalyst	V_{micro} (cm^3/g)	S_{BET} (m^2/g)	BAS (mmol/g)	LAS (mmol/g)	B/L
HZSM-5	0.15	364	0.33	0.064	5.2
HFAU40	0.33	741	0.08	0.02	4.3
HFAU15	0.32	700	0.18	0.08	2.4

Figure S3 a) ToF MS recorded at *hv* = 10.5 eV upon vanillin catalytic pyrolysis using HZSM-5 (Si/Al=25, red trace), HFAU40 (Si/Al=40, blue trace), HFAU15 (Si/Al=15, green trace) as catalysts, respectively. Reaction conditions: 577 °C; < 0.1% vanillin (*m*/*z* 152); < 0.5 bar; 20 sccm Ar. **b**) Fourier-transform infrared (FTIR) spectroscopy results for pyridine adsorption over HFAU. Brønsted acid sites (BAS) are quantified based on the 1544 cm^{-1} band.[13,14] HZSM-5 exhibits the highest BAS concentration (0.33 mmol/g) while HFAU40 has only 0.08 mmol/g. By lowering the Si/Al ratio, the BAS on HFAU15 increases to 0.18 mmol/g. **c**) ^{29}Si MAS-NMR spectra of HFAU40 and HFAU15. Both catalysts have a dominant peak at 106.8 ppm, indicating that Si atoms are mainly adjacent to Si rather than Al atoms (Q^4(0Al)) regardless of the Si/Al ratio. Indeed, Q^4(1Al) and Q^4(2Al) increases with lowering Si/Al ratio, which is interpreted as an increase of adjacent Brønsted acid sites (Figure S3**b**).[14] **d**) Scanning electron microscope images of HZSM-5, HFAU40, and HFAU15, show a similar particle size in the range of 0.7–1 μm.[14] **e**) Textural, and acid properties of different catalysts in their fresh form.[13–15] Compared to HZSM-5, HFAU has about twice as much volume and surface area. Data and figure **b**) - **e**) were partially taken from ref[14] under the CC-BY license.

<u>Phenol selectivity increase during vanillin CFP over FAU</u>

The products of vanillin catalytic pyrolysis using HZSM-5 (red trace) are mainly small molecules, such as phenol (*m*/*z* 94), fulvene (*m*/*z* 78), benzene (*m*/*z* 78), and cyclopentadiene (*m*/*z* 66). When using HFAU40 (blue trace), more primary decomposition products are observed, such as 1,2-dihydroxyl-benzaldehyde (*m*/*z* 138), guaiacol (*m*/*z* 124) and catechol (*m*/*z* 110) due to the low activity of the catalyst. Two factors are responsible for this observation: 1) HFAU40 has larger cage opening (7.4 Å) than HZSM-5 (5.5 Å). Thus, molecular diffusion is faster in HFAU40, leading to fewer secondary reactions;

2) HFAU40 has fewer active sites because of a higher Si/Al ratio (Figure S3**b** and **e**), which suppresses sequential reactions and thus secondary products. By using HFAU15 (green trace), with a higher BAS concentration (Figure S3**b** and **e**), the vanillin decarbonylation to guaiacol (*m*/*z* 124) is enhanced, which yields methyl phenol (*m*/*z* 108) and phenol (*m*/*z* 94) . The signals for small molecular products, such as benzene (*m*/*z* 78) and cyclopentadiene (*m*/*z* 66), from secondary chemistry are negligible. This aligns well with our previous findings during the guaiacol CFP experiment.[14] Also upon vanillin CFP, catechol (*m*/*z* 110), as produced from demethylation of guaiacol, is dehydroxylated to phenol, which has the highest selectivity among the products. This can be explained by inhibition of the intramolecular dehydration to fulvenone, caused by the high density of adjacent active sites (Figure S3**b**, **c**), which isolate the neighboring OH groups in catechol. This favors the dihydroxylation to yield phenol. This experiment demonstrates that guaiacol shares the same chemistry with vanillin, and the strategies, developed to optimize the guaiacol decomposition, can also be applied to vanillin.

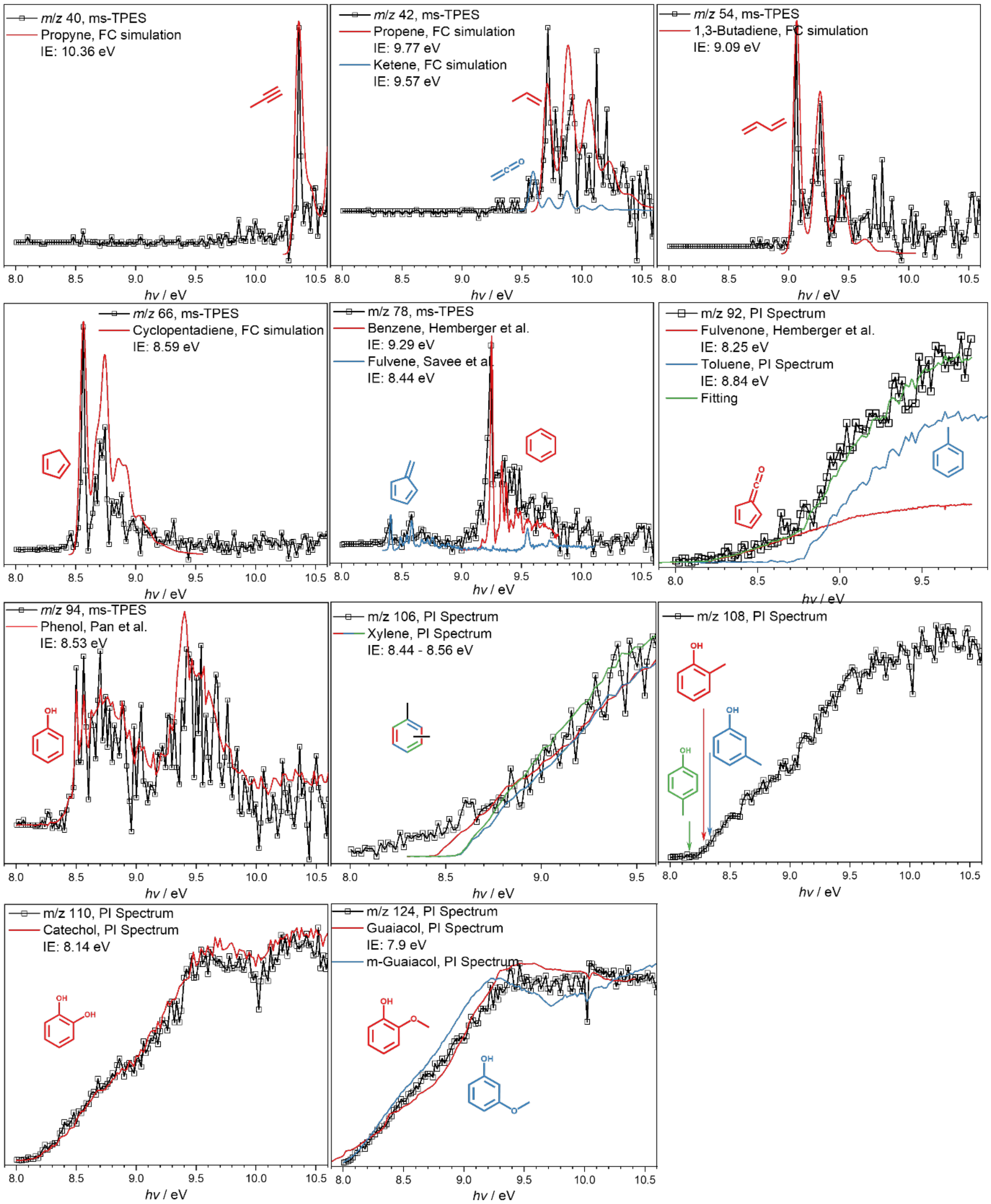

Figure S4 Photoion mass-selected threshold photoelectron spectra (ms-TPES) and photoionization spectra of syringol catalytic pyrolysis products, identified based on FC simulations combined with G4-level ionization energy (IE) calculations or experimental spectra.[9,11,12,14]

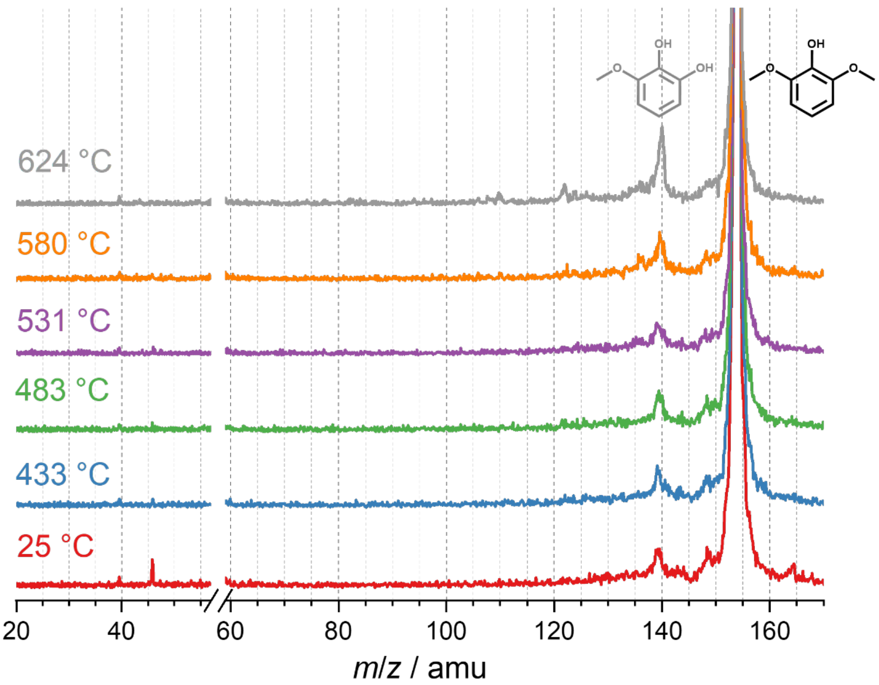

Figure S5 Time-of-flight mass spectra (ToF MS) upon syringol pyrolysis recorded at *hv* = 10.5 eV. Reaction conditions: < 0.1% syringol (*m*/*z* 154); H-ZSM-5; < 0.5 bar; 20 sccm Ar. At room temperature, syringol is present at *m*/*z* 154 accompanied by a minor peak at *m*/*z* 139, due to dissociative ionization. The peak at *m*/*z* 46 is attributed to residual ethanol from the reactor and sample container. The ethanol signal is depleted as time proceeds and becomes invisible at 433 °C. No products are observed by comparing the mass spectra at 433 °C and 25 °C. A tiny peak is seen at *m*/*z* 140 until the temperature reaches 580 °C and becomes pronounced at 624 °C, accompanied by a small peak at *m*/*z* 122. *m*/*z* 140 corresponds to the demethylation product of syringol, 3-methoxycatechol. Therefore, it is suggested that syringol does not decompose below 531 °C.

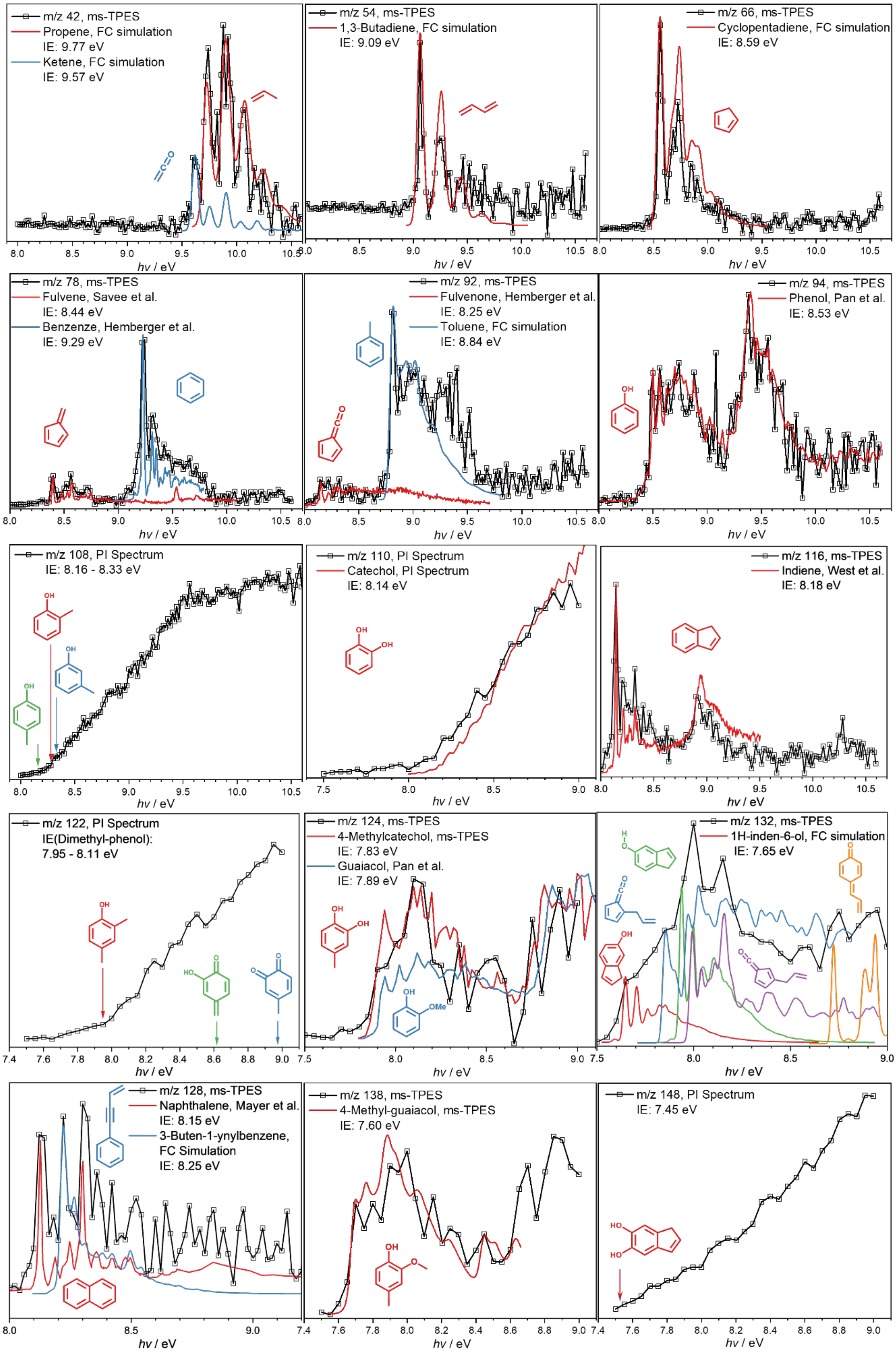

Figure S6 Photoion mass-selected threshold photoelectron spectra (ms-TPES) or photoionization spectra of products upon eugenol catalytic pyrolysis, identified based on FC simulations combined with G4 ionization energy (IE) calculations or experimental spectra.[9,11,12,14,16–18]

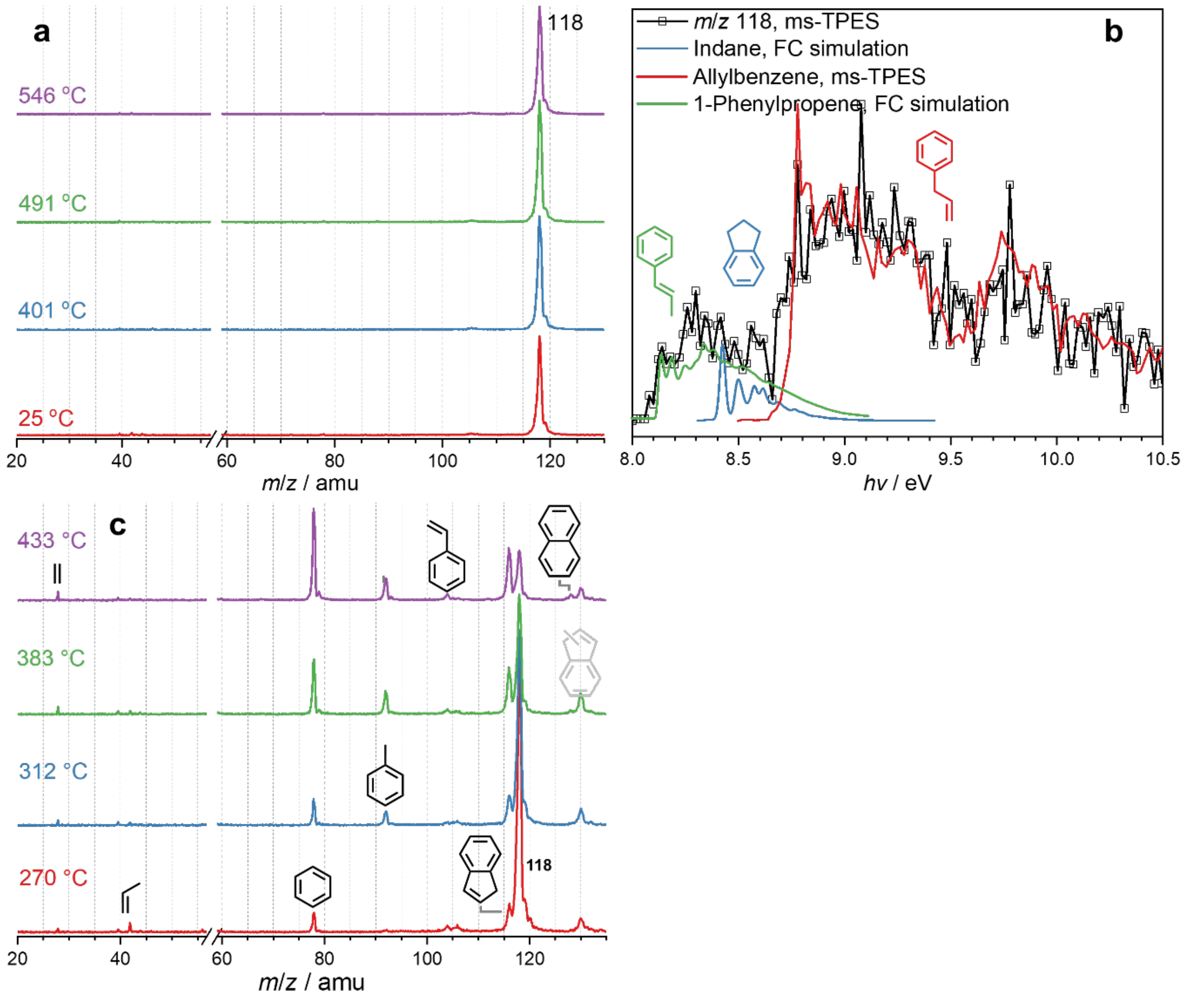

Figure S7 Allylbenzene pyrolysis. **a**) Temperature-dependent mass spectra collected at *hν*=10.5 eV. Reaction conditions: 1–2% allylbenzene (*m*/*z* 118); no catalyst; 0.11–0.16 bar; 20 sccm Ar. No obvious product peaks are observed between 25–546 °C. **b**) ms-TPES of *m*/*z* 118 (black trace), at 546 °C, with FC simulations of indane (blue trace) and 1-phenylpropene (green trace) as well as allylbenzene ms-TPES (red trace) obtained at 25 °C. *m*/*z* 118 ms-TPES shows vibrational signals between 8.7 to 10.5 eV, which agree with the reference TPES of allylbenzene. Notably, the signals in the 8.0–8.7 eV region fit the 1-phenylpropene and indane FC simulations quite well, which indicates partial isomerization at high temperature. **c**) The ring closing mechanism of the allyl group is reflected by the identification of indene and its derivates during eugenol CFP, which is best be investigated by studying the chemistry of allylbenzene. Reaction conditions: 0.8% allylbenzene (*m*/*z* 118) in Ar; HZSM-5; 0.3 bar; 20 sccm Ar. Temperature-dependent mass spectra of allylbenzene without catalyst show no obvious change in the mass spectra (a), however the ms-TPES (b) confirms rearrangement to 1-phenylpropene and indane, via ring-closing reactions at 546 °C. With the addition of HZSM-5, the reaction is initiated already at 270 °C (c) and we assign propene (*m*/*z* 42), benzene (*m*/*z* 78) and indene (*m*/*z* 116) as products (ms-TPES in Figure S8). Further investigation of the ms-TPES of *m*/*z* 118 revealed that, similar to the blank experiments, isomerization of the parent allylbenzene is observed. Besides these abundant products, we identify toluene (*m*/*z* 92) and styrene (*m*/*z* 104) at 312 °C and 433 °C, respectively. They are produced via C2 and C1 loss at the allyl group, respectively. As the temperature increases, benzene (*m*/*z* 78, Figure S8) becomes the most abundant reaction product, while fulvene is not observed. Methylation is responsible for the species at *m*/*z* 130 and 128, corresponding to a mixture of naphthalene and two benzene substituted vinyl acetylenes (Figure S8).

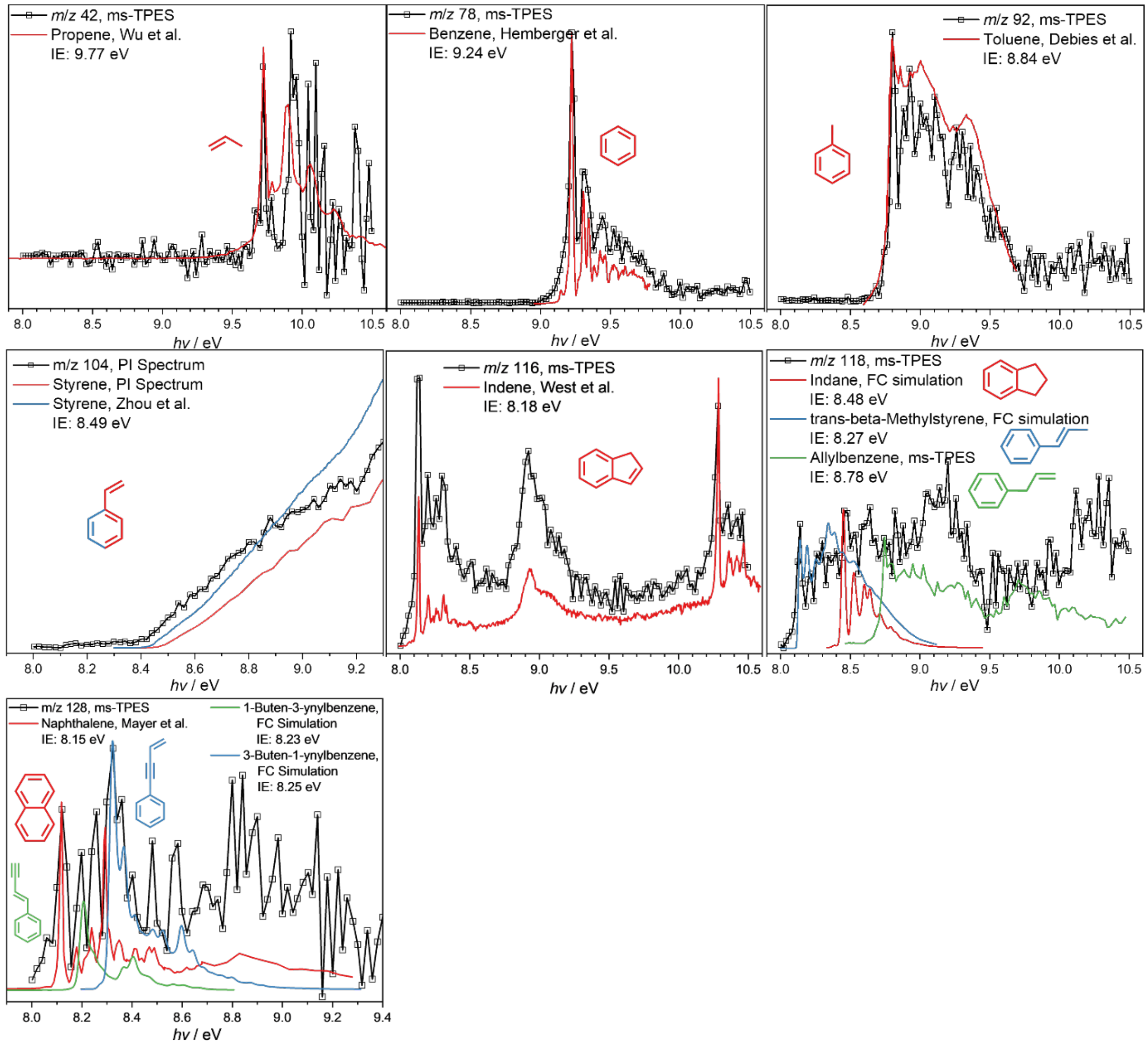

Figure S8 Photoion mass-selected threshold photoelectron spectra (ms-TPES) and photoionization spectra of products upon allylbenzene catalytic pyrolysis, identified based on FC simulations combined with G4 ionization energy (IE) calculations or reference spectra.[12,16,17,19–21]

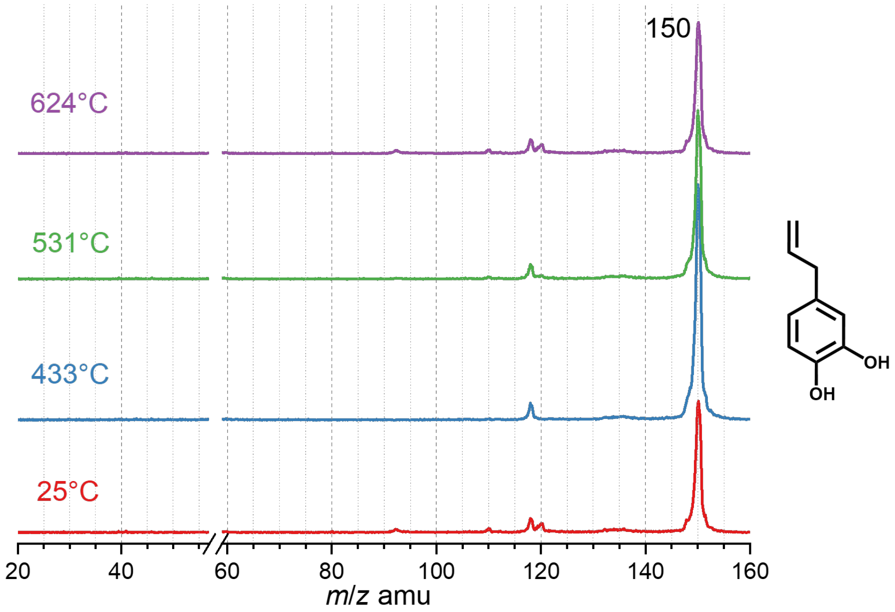

Figure S9 Temperature-dependent mass spectra upon the pyrolysis of 4-allylcatechol. Reaction conditions: 0.7–2.0% 4-allylcatechol (*m*/*z* 150); no catalyst; ~ 0.15 bar; 20 sccm Ar.

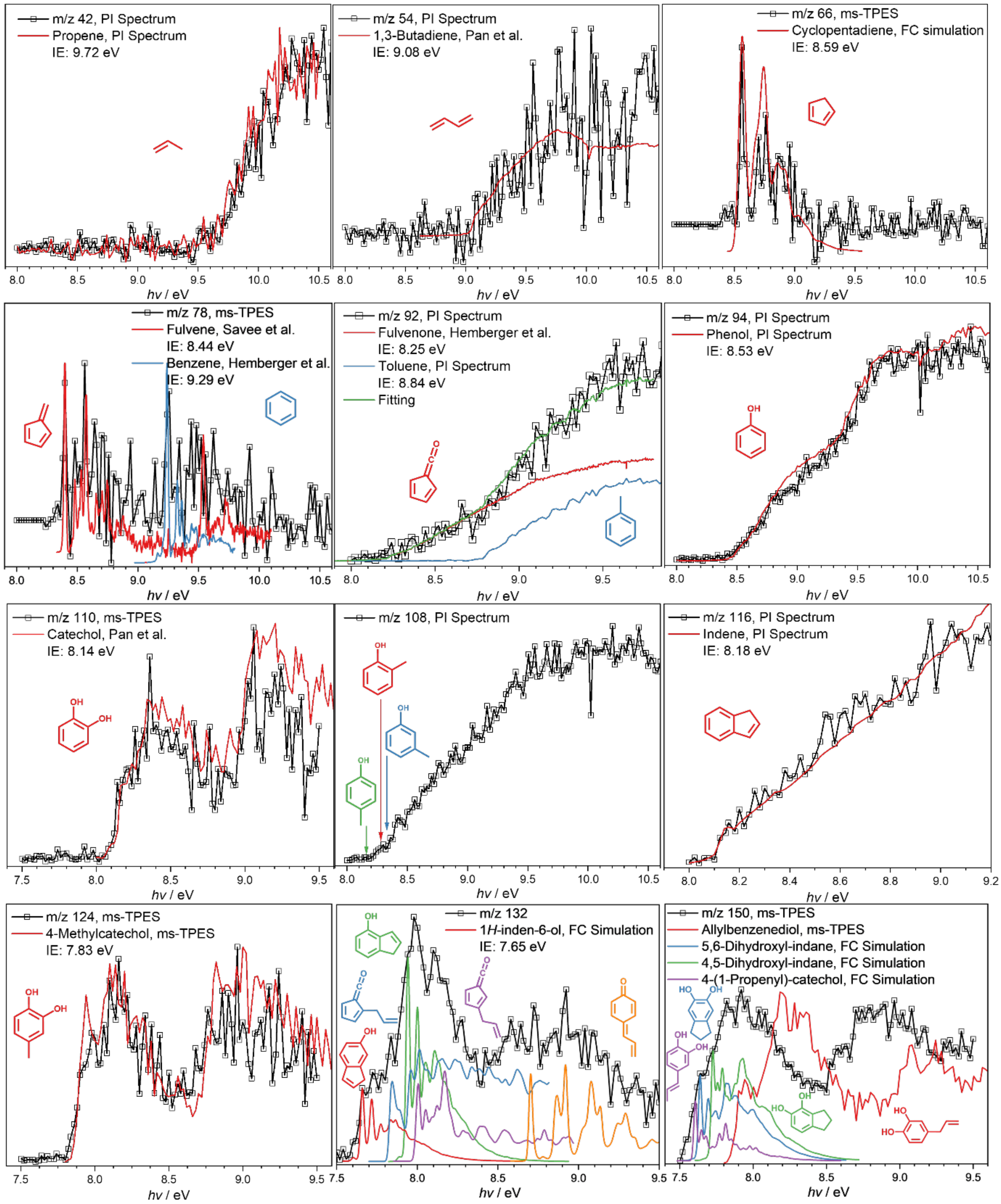

Figure S10 Photoion mass-selected threshold photoelectron spectra (ms-TPES) or photoionization spectra of products upon 4-allylcatechol catalytic pyrolysis, identified based on FC simulations combined with G4 ionization energy (IE) calculations or reference spectra.[1,9,11,12,14]

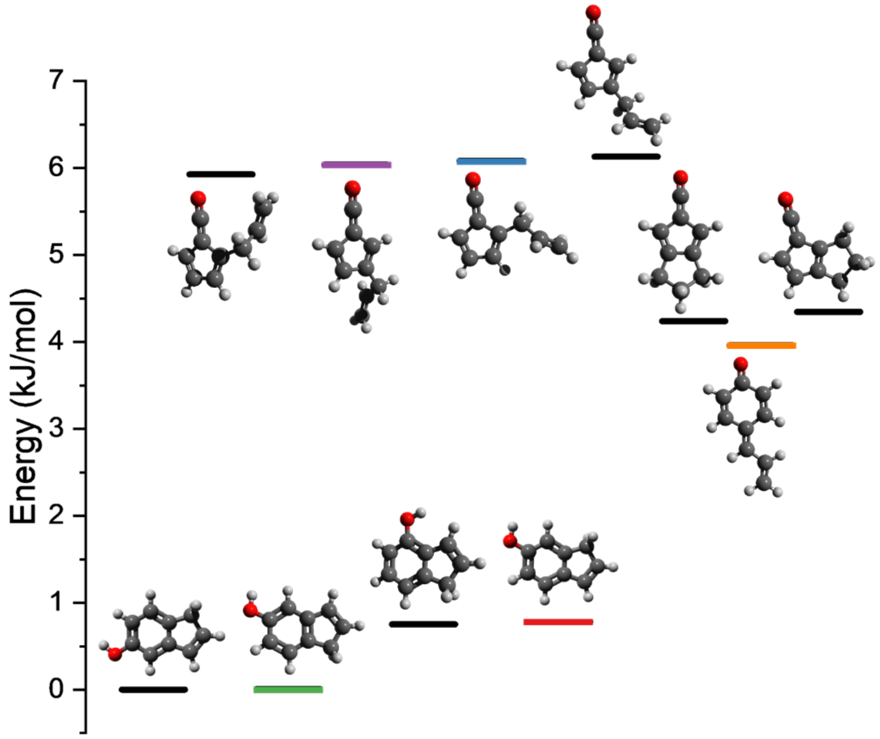

Figure S11 The internal energy comparison of *m*/*z* 132 isomers. Hydroxylindenes have similar energies. The calculated ionization energy of 1H-inden-6-ol (red line) aligns with the vibrational peak at 7.65 eV in the *m*/*z* 132 ms-TPES (Figure S10), suggesting its presence. Although the 1H-inden-6-ol is has the lowest ionization energy and thus the easiest to detect, the other hydroxyindenes may also be produced during ring closure of the allyl substituent and subsequent dehydroxylation. In addition, the methylene unit in the 5-membred ring may undergo [1,5] sigmatropic rearrangement increasing the isomeric pool. In Figure S9, no pronounced peaks of these derivatives are observed, which is probably due to their low abundance during the reaction and overlapping signals.

Fulvenone derivatives lie at ca. 3.36 kJ/mol (0.035 eV) higher in energy compared than 1H-inden-6-ol. Although the simulated spectra of the isomers plotted in purple and blue fit with experimental *m*/*z* 132 ms-TPES (Figure S10), their existence cannot be fully confirmed due to absence of distinct features in the ms-TPES.

p-Allylcyclohexadienone (orange line) lies ca. 3.18 kJ/mol (0.03 eV) higher in energy than 1H-inden-6-ol. In Figure S10, two vibrational peaks at ca. 8.7 and 8.9 eV agree with the FC simulation of *p*-allylcyclohexadienone (orange curve), which illustrates that *p*-allylcyclohexadienone is possibly produced during 4-allylcatechol catalytic pyrolysis. Those were also suggested by Shen et al. in eugenol pyrolysis, but lack of a clear spectroscopic observation.[22]

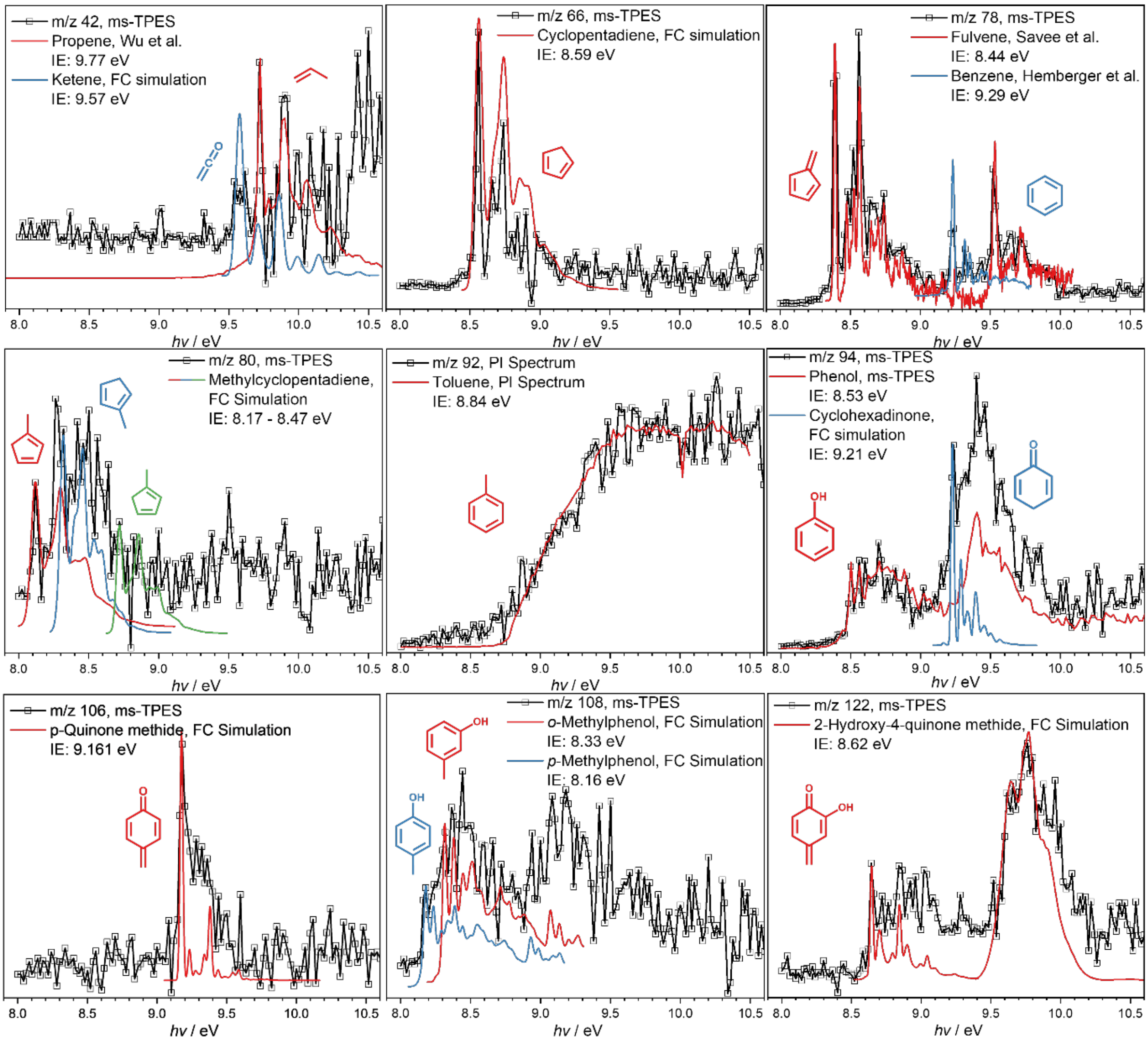

Figure S12 Photoion mass-selected threshold photoelectron spectra (ms-TPES) or photoionization spectra of products upon 4-methylcatechol catalytic pyrolysis, identified based on FC simulations combined with G4 ionization energy (IE) calculations or reference spectra.[9,12,19]

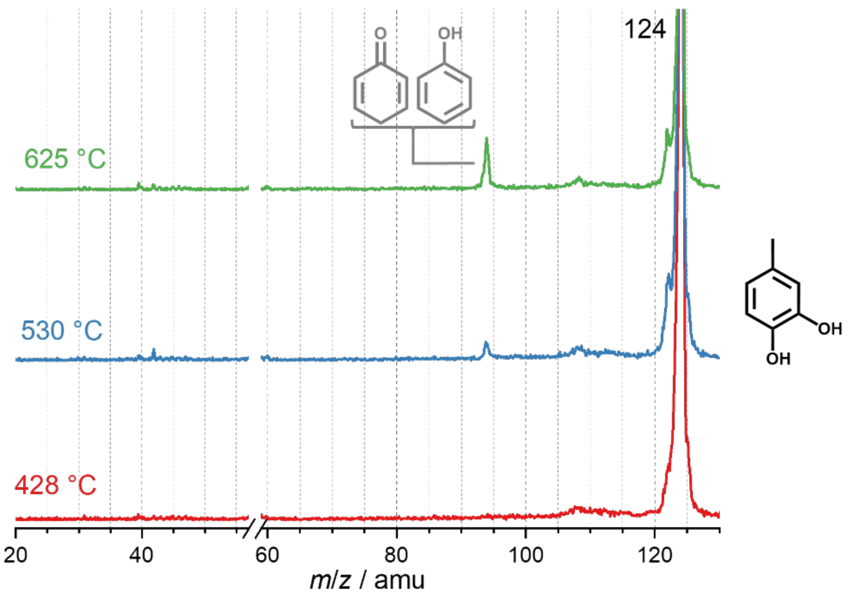

Figure S13 The temperature-dependent mass spectra upon the pyrolysis of 4-methylcatechol. Reaction conditions: < 0.1% 4-methylcatechol (*m*/*z* 124); no catalyst; ~ 0.1 bar; 20 sccm Ar.

References

1 Z. Pan, A. Puente-Urbina, A. Bodi, J. A. Van Bokhoven and P. Hemberger, *Chem. Sci.*, 2021, **12**, 3161–3169.
2 B. Sztáray, K. Voronova, K. G. Torma, K. J. Covert, A. Bodi, P. Hemberger, T. Gerber and D. L. Osborn, *J. Chem. Phys.*, 2017, **147**, 013944.
3 P. Hemberger, J. A. van Bokhoven, J. Pérez-Ramírez and A. Bodi, *Catal. Sci. Technol.*, 2020, **10**, 1975–1990.
4 P. Hemberger, A. Bodi, T. Bierkandt, M. Köhler, D. Kaczmarek and T. Kasper, *Energy Fuels*, 2021, **35**, 16265–16302.
5 P. Hemberger, Z. Pan, X. Wu, Z. Zhang, K. Kanayama and A. Bodi, *J. Phys. Chem. C*, 2023, **127**, 16751–16763.
6 A. Bodi, M. Johnson, T. Gerber, Z. Gengeliczki, B. Sztáray and T. Baer, *Rev. Sci. Instrum.*, 2009, **80**, 034101.
7 P. Hemberger, V. B. F. Custodis, A. Bodi, T. Gerber and J. A. Van Bokhoven, *Nat. Commun.*, 2017, **8**, 15946.
8 L. A. Curtiss, P. C. Redfern and K. Raghavachari, *J. Chem. Phys.*, 2007, **127**, 124105.
9 J. D. Savee, B. Sztáray, P. Hemberger, J. Zádor, A. Bodi and D. L. Osborn, *Faraday Discuss.*, 2022, **238**, 645–664.
10 X. Wu, Z. Pan, S. Bjelić, P. Hemberger and A. Bodi, *J. Anal. Appl. Pyrolysis*, 2022, **161**, 105410.
11 P. Hemberger, Z. Pan, A. Bodi, J. A. van Bokhoven, T. K. Ormond, G. B. Ellison, N. Genossar and J. H. Baraban, *ChemPhysChem*, 2020, **21**, 2217–2222.
12 P. Hemberger, X. Wu, Z. Pan and A. Bodi, *J. Phys. Chem. A*, 2022, **126**, 2196–2210.
13 C. W. P. Pare, P. Rzepka, P. Hemberger, A. Bodi, R. Hauert, J. A. Van Bokhoven and V. Paunović, *ACS Catal.*, 2024, **14**, 463–474.
14 Z. Pan, A. Puente-Urbina, S. R. Batool, A. Bodi, X. Wu, Z. Zhang, J. A. Van Bokhoven and P. Hemberger, *Nat. Commun.*, 2023, **14**, 4512.
15 S. R. Batool, V. L. Sushkevich and J. A. Van Bokhoven, *ACS Catal.*, 2024, **14**, 678–690.
16 B. West, A. Sit, A. Bodi, P. Hemberger and P. M. Mayer, *J. Phys. Chem. A*, 2014, **118**, 11226–11234.
17 P. M. Mayer, V. Blanchet and C. Joblin, *J. Chem. Phys.*, 2011, **134**, 244312.
18 Z. Pan, A. Bodi, J. A. van Bokhoven and P. Hemberger, *Phys. Chem. Chem. Phys.*, 2022, **24**, 21786–21793.
19 X. Wu, Z. Zhang, Z. Pan, X. Zhou, A. Bodi and P. Hemberger, *Angew. Chem. Int. Ed.*, 2022, **61**, e202207777.
20 T. P. Debies and J. W. Rabalais, *J. Electron Spectrosc. Relat. Phenom.*, 1972, **1**, 355–370.
21 Z. Zhou, M. Xie, Z. Wang and F. Qi, *Rapid Commun. Mass Spectrom.*, 2009, **23**, 3994–4002.
22 Y. Shen, X. Liu, H. Ren, X. Xiao, R. Sun, H. Liu, J. Lu, C. Huang, L. Zhao, Z. Zhou and F. Qi, *J. Anal. Appl. Pyrolysis*, 2024, **177**, 106317.
